# Karyotype differentiation in 19 species of river loach fishes (Nemacheilidae, Teleostei): extensive variability associated with rDNA and heterochromatin distribution and its phylogenetic and ecological interpretation

**DOI:** 10.1186/s12862-015-0532-9

**Published:** 2015-11-14

**Authors:** Alexandr Sember, Jörg Bohlen, Vendula Šlechtová, Marie Altmanová, Radka Symonová, Petr Ráb

**Affiliations:** Laboratory of Fish Genetics, Institute of Animal Physiology and Genetics, Czech Academy of Sciences, Rumburská 89, Liběchov, 277 21 Czech Republic; Department of Genetics and Microbiology, Faculty of Science, Charles University in Prague, Viničná 5, 128 44 Prague 2, Czech Republic; Department of Ecology, Faculty of Science, Charles University in Prague, Viničná 7, 128 44 Prague 2, Czech Republic; Research Institute for Limnology, University of Innsbruck, Mondseestraße 9, A-5310 Mondsee, Austria

**Keywords:** Fish cytotaxonomy, Karyotype variability vs. 2n uniformity, FISH, 45S - 5S ribosomal genes, Chromosome banding, Pericentric inversion, Robertsonian translocation, Effective population size

## Abstract

**Background:**

Loaches of the family Nemacheilidae are one of the most speciose elements of Palearctic freshwater ichthyofauna and have undergone rapid ecological adaptations and colonizations. Their cytotaxonomy is largely unexplored; with the impact of cytogenetical changes on this evolutionary diversification still unknown. An extensive cytogenetical survey was performed in 19 nemacheilid species using both conventional (Giemsa staining, C- banding, Ag- and Chromomycin A_3_/DAPI stainings) and molecular (fluorescence in situ hybridization with 5S rDNA, 45S rDNA, and telomeric (TTAGGG)_n_ probes) methods. A phylogenetic tree of the analysed specimens was constructed based on one mitochondrial (*cytochrome b*) and two nuclear (*RAG1*, *IRBP*) genes.

**Results:**

Seventeen species showed karyotypes composed of 2n = 50 chromosomes but differentiated by fundamental chromosome number (NF = 68–90). *Nemachilichthys ruppelli* (2n = 38) and *Schistura notostigma* (2n = 44–48) displayed reduced 2n with an elevated number of large metacentric chromosomes. Only *Schistura fasciolata* showed morphologically differentiated sex chromosomes with a multiple system of the XY_1_Y_2_ type. Chromomycin A_3_ (CMA_3_)- fluorescence revealed interspecific heterogeneity in the distribution of GC-rich heterochromatin including its otherwise very rare association with 5S rDNA sites. The 45S rDNA sites were mostly located on a single chromosome pair contrasting markedly with a pattern of two (*Barbatula barbatula*, *Nemacheilus binotatus*, *N. ruppelli*) to 20 sites (*Physoschistura* sp.) of 5S rDNA. The cytogenetic changes did not follow the phylogenetic relationships between the samples. A high number of 5S rDNA sites was present in species with small effective population sizes.

**Conclusion:**

Despite a prevailing conservatism of 2n, Nemacheilidae exhibited a remarkable cytogenetic variability on microstructural level. We suggest an important role for pericentric inversions, tandem and centric fusions in nemacheilid karyotype differentiation. Short repetitive sequences, genetic drift, founder effect, as well as the involvement of transposable elements in the dispersion of ribosomal DNA sites, might also have played a role in evolutionary processes such as reproductive isolation. These remarkable dynamics of their genomes qualify river loaches as a model for the study of the cytogenetic background of major evolutionary processes such as radiation, endemism and colonization of a wide range of habitats.

**Electronic supplementary material:**

The online version of this article (doi:10.1186/s12862-015-0532-9) contains supplementary material, which is available to authorized users.

## Background

Cypriniformes, the largest order of freshwater fishes globally, is composed of two highly diverse Palearctic superfamilies – Cyprinoidea and Cobitoidea [1, 2]. Cobitoidea, or “loaches”, are a group of small benthic fishes which are one of the most common elements of Eurasian freshwater ichthyofauna. To date, Cobitoidea includes about 1100 species, currently recognized in ten families [3], and yet only representatives of Cobitidae, Botiidae, Catostomidae and Vaillantellidae have so far been studied cytogenetically. Several cases of highly diverse karyotypes and polyploidy have been discovered in the first three families, although not in Vaillantellidae [4]. The Botiidae family consists of two subfamilies differing in ploidy levels (one diploid and one tetraploid) [5]. In Cobitidae, several independent polyploidization events occurred [6, 7], in some cases after hybridization, leading to an asexual mode of reproduction [8–10]. From these limited data we can see that cytogenetic changes might have played an important role in the evolution of loaches and it remains an open question as to whether this is also true for the remaining cobitoid lineages.

With nearly 600 recognized species in 46 genera [3], Nemacheilidae, or “river loach”, represents the most diverse family of loach fishes, as well as being the most widespread with a distribution area ranging continuously from Portugal to Japan, and from most Siberian rivers to Java [11]. Importantly, river loaches are also very abundant within this enormous distribution area, occurring in virtually all rivers in Europe and Asia. On the other hand, their distribution pattern varies considerably; while some species are geographically very restricted, others are widely distributed, a feature often found even within the same genus, e.g., *Schistura* [3]. Additionally, Nemacheilidae have colonized an unusual variety of habitats including standing swamps, torrential rapids, major rivers, small forest streams, caves and lakes. Their ecological diversity is further illustrated by them being both the highest (above sea level) and the lowest (below ground level) freshwater fish in the world [3]. All these characteristics make Nemacheilidae a vital model for evolutionary study and our candidate group with which to evaluate the impact of cytogenetic changes on their diversity.

Despite the vast biodiversity within Nemacheilidae, the cytogenetics and cytotaxonomy of this group remain poorly explored. Giemsa-stained chromosomes have been studied in only 24 species [7, 12–14] and banding techniques were performed solely in the single species *Barbatula barbatula* [15] while no molecular cytogenetics had previously been applied. From this limited data, karyotypes of most analysed species display the stable diploid chromosome number 2n = 50, while interspecific karyotype variability in the number of chromosomal arms (Nombre Fundamental, NF) is apparent (see, e.g., [15–17]). In some species, intraspecific variability in 2n and karyotype composition has also been documented [17–20]. Polyploidy has been recorded only in one species *B. ‘barbatula’* (2n = 3x = 75) [21]. The scarce available data does indicate the extensive but unexplored cytogenetic diversity of nemacheilid loaches.

The aim of this study is to assess cytogenetic variability within the Nemacheilidae family using conventional and molecular chromosome markers and to evaluate these data with regards to the evolutionary processes behind morphological and ecological diversification. A representative sampling of 19 species from eleven genera were used to investigate karyotypes, heterochromatin distribution and chromosomal characteristics of both rDNA classes and (in some cases) the telomeric sequence motif (TTAGGG)_n_. All cytogenetic characteristics were mapped onto a phylogenetic tree based on molecular analyses of one mitochondrial and two nuclear genes.

## Methods

### Animals

**Fig. 1:**
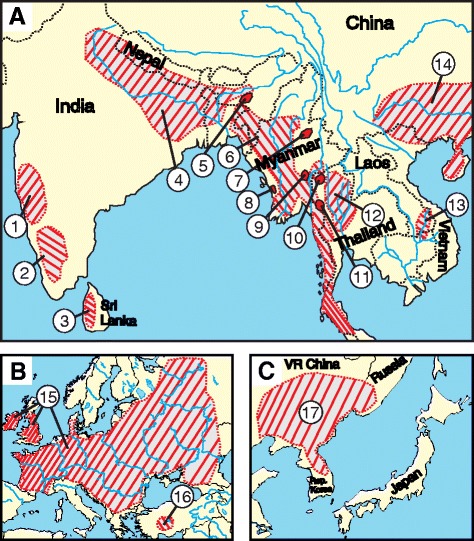
Distribution areas of the investigated species of Nemacheilidae. **a** Asia, **b** Europe, **c** China. 1 – *N. ruppelli*, 2 – *M. guentheri*, 3 – *S. notostigma*, 4 – *S. corica* and *S. savona*, 5 – *P. elongata*, 6 – *P. zonalternans*, 7 – *P. lucidorsum*, 8 – *S. hypsiura*, 9 – *P. sp.* and *P. brevis*, 10 – *S. pridii*, 11 – *P. pictilis*, 12 – *N. binotatus*, 13 – *S. bolavensis*, 14 – *S. fasciolata*, 15 – *B. barbatula*, 16 – *S. lendlii*, 17 – *L. costata*

Fifty-two individuals belonging to 19 different nemacheilid species were analysed (Table 1). Their distribution areas are specified in Fig. 1 and references for taxonomic identification are given in Additional file 1: Supplementary Methods 1. All analysed specimens were obtained from ornamental fish trade, from a commercial fish farm or from private aquarium fish breeders. All experimental procedures involving fishes were approved by the Institutional Animal Care and Use Committee of the IAPG AS CR, according with directives from the State Veterinary Administration of the Czech Republic, permit number 217/2010, and by permit number CZ 02386 from the Ministry of Agriculture of the Czech Republic. Voucher specimens are deposited to the fish collection of the Laboratory of Fish Genetics, IAPG, CAS, Liběchov.

**Table 1:** Species under study, their sex, origin and geographical distribution

Species	Individuals	Source (country, province, river basin)	Distribution
*Barbatula barbatula* (Linnaeus, 1758)	3	Czech Republic, Středočeský kraj, Elbe	widespread (Europe, Asia)
*Lefua costata* (Kessler, 1876)	2♀	Republic of Korea, Gangwon, Geojin	widespread (Korea, China)
*Mesonoemacheilus guentheri* (Day, 1867)	1♂, 1♀	Ornamental fish trade	moderately widespread (southern India)
*Nemacheilus binotatus* (Smith, 1933)	1♂, 1♀	Ornamental fish trade	moderately widespread (Thailand)
*Nemachilichthys ruppelli* (Sykes, 1839)	1♂, 1♀	Ornamental fish trade	moderately widespread (southern India)
*Paracanthocobitis pictilis* (Kottelat, 2012)	2♀	Ornamental fish trade	endemic to Ataran river (Myanmar)
*Paracanthocobitis zonalternans* (Blyth, 1860)	1 ♂1♀	Myanmar, no details known	widespread (Bangladesh to Malaysia)
*Petruichthys brevis* (Boulenger, 1893)	1♂, 1♀ + 1	Ornamental fish trade	endemic to Inle Lake (Myanmar)
*Physoschistura elongata* (Sen & Nalbant, in Singh, Sen, Bănărescu & Nalbant, 1982)	2	Ornamental fish trade	endemic to Shilling county (northeast India)
*Physoschistura* sp.	2	Myanmar, Shan, Salween	endemic to surrounding of Inle Lake (Myanmar)
*Pteronemacheilus lucidorsum* (Bohlen & Šlechtová, 2011)	1♂, 1♀	Myanmar, Shan, Irrawaddy	endemic to upper Myitnge river basin (Myanmar)
*Schistura bolavenensis* (Kottelat, 2000)	3	Laos: Champasak, Mekong	moderately spread (Bolaven plateau, Laos)
*Schistura corica* (Hamilton, 1822)	1♂, 3♀	Ornamental fish trade	widespread (northern India, Bangladesh)
*Schistura fasciolata* (Nichols and Pope, 1927)	2♂, 1♀	Ornamental fish trade	widespread (southern China and northern Vietnam)
*Schistura hypsiura* (Bohlen, Šlechtová & Udomritthiruj, 2014)	1♂, 1♀ +3	Ornamental fish trade	endemic to southern Rakhine state (Myanmar)
*Schistura notostigma* (Bleeker, 1863)	6	Ornamental fish trade	endemic (Sri Lanka)
*Schistura pridii* (Vidthayanon, 2003)	2	Ornamental fish trade	local endemic (northern Thailand)
*Schistura savona* (Hamilton, 1822)	3	Ornamental fish trade	widespread (northern India, Bangladesh)
*Seminemacheilus lendlii* (Hankó, 1924)	1♂, 1♀	Turkey, Anatolia, no details known	endemic to southeast Anatolia (Turkey)

### Chromosome preparation and analysis of constitutive heterochromatin

Mitotic chromosomes were obtained from regenerating fin tissue by the technique described by Völker et al. [22] and Völker and Ráb [23], with slight modifications (see Additional file 1: Supplementary Methods 2). For conventional cytogenetic analysis, chromosomes were stained with 5 % Giemsa solution (pH 6.8) (Merck, Darmstadt, Germany). Selected slides were destained in methanol:acetic acid fixation (see above) and re-used for the other techniques. For fluorescence in situ hybridization (FISH), slides were dehydrated in an ethanol series (70, 80 and 96 %, 3 min each) and stored in a freezer (−20 °C). Visualization of the constitutive heterochromatin was done by C-banding according to Haaf and Schmid [24] using 4′,6-diamidino-2-phenolindole (DAPI) (Sigma, St. Louis, MO, USA) counterstaining. Fluorescence staining was performed sequentially or in separate experiments by GC-specific fluorochrome Chromomycin A_3_ (CMA_3_) (Sigma-Aldrich) [25] and AT-specific fluorochrome DAPI (Sigma-Aldrich) [26], following Mayr et al. [27] and Sola et al. [28]. In *P. elongata*, a silver staining technique was employed according to Howell and Black [29]. At least ten metaphases per specimen were analysed, in some cases sequentially. In a few cases, metaphases with incomplete 2n were selected (see Figs. 6i and 7a; Additional file 2: Figure S1K), but were sufficient enough to present the required features. Chromosome morphology was classified according to Levan et al. [30], but modified as m – metacentric, sm – submetacentric, st – subtelocentric, a – acrocentric, where st and a chromosomes were scored as uniarmed, together in one category.

### DNA isolation and probe preparation

Whole genomic DNA was extracted from fin tissue using the conventional phenol-chloroform-izoamylalcohol method [31] using PhaseLock Eppendorf tubes (5PRIME, Gaithersburg, USA) to prevent protein contamination, or the Qiagen DNAeasy Blood & Tissue Kit (Qiagen, Hilden, Germany). rDNA fragments were obtained by polymerase chain reaction (PCR) using previously described primers (see Additional file 3: Table S1; for PCR conditions see Additional file 1: Supplementary Methods 3). The resulting PCR products were purified using QIAquick PCR purification Kit (Qiagen), with multiple bands being electrophoresed in 0.8 % agarose gels and purified using QIAquick Gel Extraction Kit (Qiagen). DNA fragments were cloned to pDrive Cloning Vector (Qiagen) and transformed into QIAGEN EZ Competent Cells (Qiagen). Selected recombinant plasmids were isolated by QIAprep Spin Miniprep Kit (Qiagen) and sequenced in both strands by Macrogen (South Korea, Netherlands). Chromatograms of obtained sequences were verified and assembled using SeqMan Pro 10.1.2 (LaserGene, DNASTAR, Madison, Wl.). The resulting consensus sequences were confirmed using NCBI BLAST/N analysis [32] and selected clones used to construct FISH probes.

Probes were labelled by PCR with biotin-16-dUTP (Roche, Mannheim, Germany) or digoxigenin-11-dUTP (Roche). For each slide 200 ng of 5S rDNA, 200 ng of 45S rDNA and 25 μg of sonicated salmon sperm DNA (Sigma-Aldrich) were added and the resulting probe precipitated in 96 % ethanol, washed in 70 % ethanol, air-dried and re-dissolved in hybridization buffer (50 % formamide, 10 % dextran sulphate, 2× SSC, 0.04 M NaPO_4_ buffer, 0.1 % SDS, Denhardt reagens, see [33]) to give a final concentration of 25 ng/μl for each rDNA probe.

For telomeric FISH, non-templated PCR with primers (TTAGGG)_5_ and (CCCTAA)_5_ was carried out according to Ijdo et al. [34]. The amplified product was labelled using Nick Translation Mix (Abbot Molecular, Illinois, USA) with biotin-16-dUTP, taking 3–4 h to reach optimal probe size (100–500 bp).

### FISH analysis

FISH was carried out according to Cremer et al. [35] with several modifications. Briefly, dehydration in an ethanol series (70, 80 and 96 %, 3 min each) was followed by thermal aging for 1–2 h at 37 °C and 30 min at 60 °C. Prior to hybridization, the chromosomes were treated with RNase A (200 μg/ml in 2× SSC) (Sigma-Aldrich) for 90 min at 37 °C in a humid chamber and digested with pepsin (50 μg/ml in 10 mM HCl, 3 min, 37 °C). Slides were subsequently denatured in 75 % formamide (pH 7.0) (Sigma-Aldrich) in 2× SSC at 74 °C for 3 min, and then immediately cooled and dehydrated in 70 % (cold), 80 % and 96 % (RT) ethanol. The hybridization mixture was denatured at 86 °C for 6 min and immediately chilled on ice for 10 min. 10–20 μl of probe mixture was applied to a denatured slide and hybridization was performed overnight at 37 °C in a dark humid chamber. Post-hybridization washes were done twice in 50 % formamide in 2× SSC (pH 7.0) at 42 °C for 5 min and three times in 1× SSC at 42 °C (7 min each) before equilibration washing in 2× SSC at RT for 20 s. Prior to probe detection 500 μl of 3 % BSA (Vector Labs, Burlington, Canada) in 4× SSC in 0,01 % Tween 20 was dropped onto the slide (at 37 °C for 20 min) as a blocking treatment. Probes were detected by Anti-Digoxigenin-Rhodamine (Roche) and Streptavidin-FITC (Invitrogen Life Technologies, San Diego, CA, USA) along with Anti-Digoxigenin-Fluorescein (Roche) and Streptavidin-Cy3 (Invitrogen Life Technologies) to exclude any artificial results (influenced e.g., by the type of applied antibody). Experiments with altered labelling (biotin for 45S and digoxigenin for 5S rDNA) were included to verify the observed patterns. All rDNA FISH pictures presented here are pseudocoloured in red for the 45S rDNA probe and in green for the 5S rDNA.

The slides were incubated with antibodies at 37 °C for 60 min in a dark humid chamber, washed four times (7 min each) in 4× SSC in 0.01 % Tween (pH 7.0) at 42 °C and the chromosomes then counterstained with DAPI in mounting medium (Cambio, Cambridge, United Kingdom), covered and sealed with a coverslip.

To enhance telomeric FISH signals, tyramid signal amplification (TSA) was performed using a kit with tyramide conjugated with Alexa 488 fluorochrome (Invitrogen Life Technologies).

After image processing FISH slides selected for fluorescence banding and/or C-banding were washed in 4× SSC in 0.01 % Tween (pH 7.0) and dehydrated in an ethanol series.

### Microscopy and image analysis

Giemsa-stained chromosomes and FISH images were inspected using a Provis AX70 Olympus microscope with a standard fluorescence filter set. FISH images were captured under immersion objective 100× with a black and white CCD camera (DP30W Olympus) for each fluorescent dye using Olympus Acquisition Software. The digital images were then pseudocoloured (blue for DAPI, red for Rhodamine or Cy3, green for FITC or Alexa488) and superimposed with MicroImage software (Olympus, version 4.0). FISH karyotype images were optimized and arranged using Adobe Photoshop, version CS6. Karyotypes from Giemsa-stained and C-banded images were arranged in IKAROS (Metasystems) software.

### Phylogenetic analyses

Phylogenetic hypothesis was based on the analyses of three molecular markers: mitochondrial *cytochrome b* (*cyt b*), *recombination-activating gene 1* (*RAG1*) and *interphotoreceptor retinoid-binding protein* (*IRBP*). The primers and PCR reaction protocols for *cyt b* and *RAG1* followed Šlechtová et al. [5, 36], and Chen et al. [37] for the *IRBP* amplification (for details, see Additional file 1: Supplementary Methods 4). The same sets of PCR primers were used for sequencing (summarized for all genes in Additional file 3: Table S1). All three genes were sequenced for each of the 39 analysed specimens of Nemacheilidae.

Chromatograms were edited and assembled using SeqMan Pro 10.1.2 (LaserGene, DNASTAR). The sequences were aligned in BioEdit 7.0.5.3 [38] and evaluated based on their amino acid translation.

Prior to the phylogenetic analyses, the congruence among the three gene partitions was assessed using the incongruence length difference (ILD) test [39] with 1000 replication as implemented in PAUP 4.0b10 [40]. Since the test did not reveal any significant conflict (see the Results), all three datasets could be concatenated into a single matrix.

Alignments of all three genes were concatenated into a single 2998 bp dataset (1124 bp of *cyt b*, 974 bp of *RAG1* and 900 bp of *IRBP*) and 40 individuals (39 Nemacheilidae plus 1 outgroup). All sequences but one (*cyt b* sequence of *Botia lohachata*) are original data and were deposited in GenBank [41] under the accession numbers [KP738491 - KP738609] (see Additional file 4: Table S2).

Phylogenetic analysis of the concatenated dataset was performed using the partitioned Bayesian inference in MrBayes 3.2.2 [42]. The dataset was partitioned by genes and codon positions, involving in total nine partitions. The analysis was set to six Metropolis Coupled Markov Chains Monte Carlo (MCMCMC) with default heating conditions, searching the tree space for 5 milion generations under the GTR + G + I settings for each partition, in two runs, starting with random trees and a sampling frequency of each 100 generations. The log-likelihood score distribution was examined to determine the burn-in values. The first 1000 trees were discarded as burn-in and the remaining ones were used to build a 50 % majority rule consensus tree and statistical support of clades was assessed by posterior probabilities.

## Results

### Sequence analysis of *RAG1*, *IRBP* and *cyt b*

The *RAG1*, *cytochrome b* and *IRBP* datasets consisted of 974 (30 % of variable positions), 1124 (44 % v.p.) and 900 bp (35 % v.p.), respectively. The ILD test did not reject the null hypothesis about the homogeneity of any of the analysed datasets: *P* = 0.94 for *RAG1* vs. *cyt b*, *P* = 0.71 for *cyt b* vs. *IRBP* and *P* = 0.14 for *RAG1* vs. *IRBP*. Therefore the data were concatenated into a single dataset for the further analysis, altogether providing a dataset of 2998 bp.

In the final phylogeny all analysed species were identified as monophyletic and well-separated lineages. The topology shows a prominent basal split into one major clade that contains *Nemacheilus binotatus* from northern Thailand plus all samples from Myanmar, India, Sri Lanka and Turkey and a second major clade that is composed from all samples from China, Laos, Europe and Korea. Within the first major clade, four subclades are visible: the first containing *N. binotatus*, the second *Schistura savona* and both species of *Paracanthocobitis*, the third solely *Nemachilichthys ruppelli* and the fourth containing all remaining samples from the genera *Mesonoemacheilus, Schistura, Physoschistura, Seminemacheilus, Pteronemacheilus* and *Petruichthys*. Within the second major clade, three subclades are visible: the first containing *Lefua costata* from Korea*,* the second *B. barbatula* from Europe and the third with two species of *Schistura* from Laos and China.

### Sequence analysis of 5S and 28S rDNA

PCR amplification of 28S rDNA resulted consistently in a fragment 300 bp in size, containing partial sequence of 28S rRNA coding region. Sequences for *P. elongata*, *S. bolavenensis, S. corica, S. fasciolata* as well as for *Botia almorhae* (from related family Botiidae) were deposited in GenBank [41] (see Additional file 5: Table S3). For 5S rDNA, a high degree of variability, both in length as well as in number of putative 5S rDNA fragments was observed among the analysed species, so sequenced fragments from *Esox lucius* (300 bp) and *B. almorhae* (500 bp) were used for constructing the FISH probe. The sequence of 5S rDNA fragment from *E. lucius* was verified in GenBank [EF514228]. The sequence of 5S rDNA from *B. almorhae* (deposited in GenBank; see Additional file 5: Table S3) contained a partial sequence of the 5S rDNA coding region (83 bp) and a putative NTS (non-transcribed spacer). For detailed analysis of nemacheilid 5S rDNA, we selected 200 and 600 bp PCR fragments from two specimens of *S. pridii*. Thirteen clones were sequenced and verified in BLAST/N and also searched against the Repbase database at the Genetic Information Research Institute (GIRI) [43] for the presence of transposable elements (TEs) or other repetitive sequences. Indeed, each cloned sequence contained - next to the 71 bp of the 5S rRNA gene coding region - a putative NTS (85 bp or 475 bp) containing a fragment (54 bp) of L1-2_DR non-long terminal repeat (non-LTR) retrotransposon (RTE) at the 3′end (Additional file 6: Figure S2). The differences between both PCR fragments were thus in the length of the putative NTS and in the distance of the RTE fragment from the 5S rRNA coding region. No such association between TEs and rDNA loci was observed in the 5S rDNA of *B. almorhae* or in the 28S rDNA fragments characterized in this study.

### Cytogenetic characteristics

**Fig. 2:**
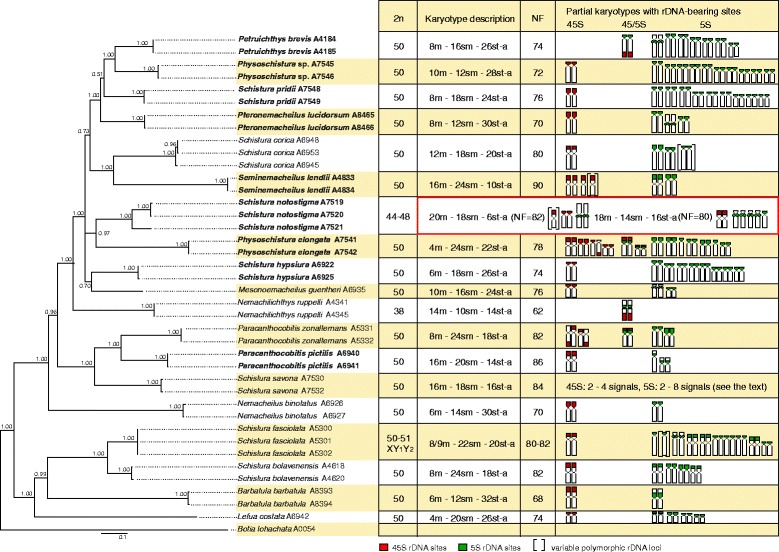
Phylogenetic relationships and karyotype characteristics of inspected nemacheilids. 2n, karyotype characteristics, FN, 45S and 5S rDNA patterns are plotted onto phylogenetic tree obtained by Bayesian analysis based on the mitochondrial (*cytochrome b*) and nuclear (*RAG1*, *IRBP*) genes. Idiograms represent partial karyotypes with chromosomes bearing 45S rDNA (*red signals*) and 5S rDNA (*green signals*). Polymorphic rDNA sites are in brackets. Note higher numbers of 5S rDNA sites in the majority of endemic species (whose taxonomic names are in bold italics). Note: in *P. elongata*, only the karyotype version from one individual is presented, to avoid confusion due to the high number and variability of rDNA sites in this species

**Fig. 3:**
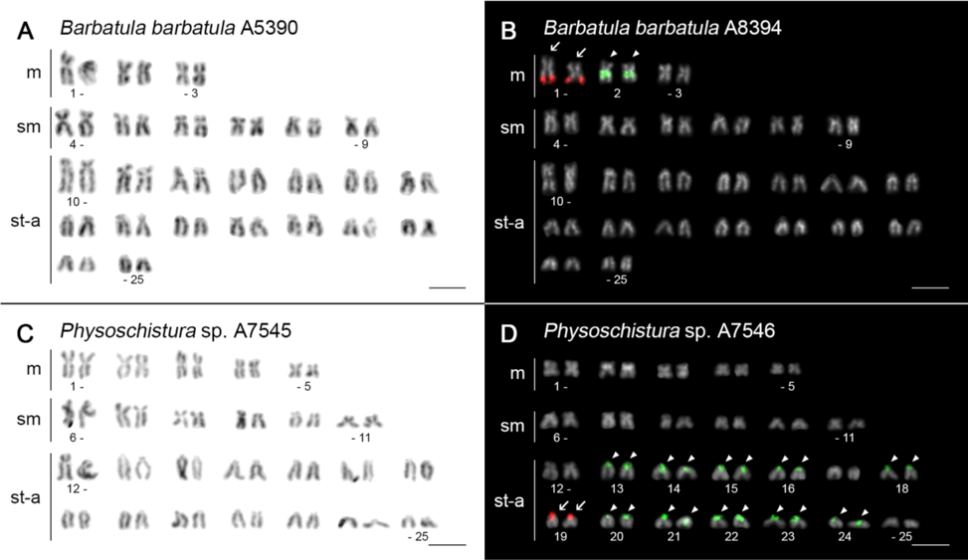
Karyotypes of selected nemacheilid species after Giemsa staining and dual-colour (5S/45S) rDNA FISH. Giemsa-stained karyotypes (*left column*) and dual-colour FISH (*right column*) with 45S rDNA (*red, arrows*) and 5S rDNA (*green, arrowheads*) probes on (**a**, **b**) *B. barbatula*, (**c**, **d**) *P.* sp*.* The FISH chromosomes were counterstained with DAPI and the images were converted to grayscale. Bar = 10 μm

**Fig. 4:**
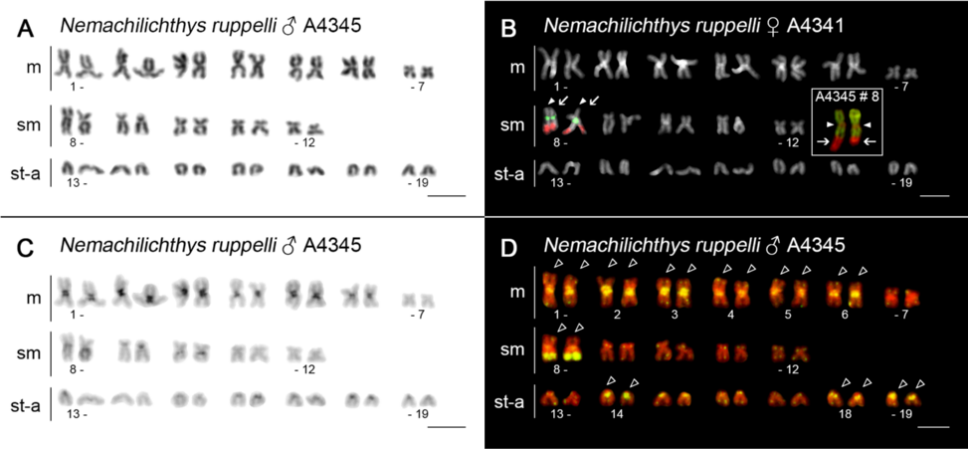
Karyotypes of *N. ruppelli* after different cytogenetic protocols. **a** conventional Giemsa staining, **b** dual-colour FISH with 45S rDNA (*red, arrows*) and 5S rDNA (*green, arrowheads*) probes, **c** C-banding and **d** FISH with telomeric (TTAGGG)_n_ probe. For better contrast, pictures were pseudocoloured in green (telomeric probe) and red (DAPI). Inset (**b**) – chromosome pair 8 bearing CMA_3_^+^ sites coinciding with both 45S (*arrow*) and 5S rDNA (*arrowhead*) sites. For better contrast, pictures were pseudocoloured in red (CMA_3_^+^) and green (DAPI). Note the prominent pericentromeric heterochromatin in metacentric chromosome pairs 1–6 (**b**) and an almost equivalent intensity of percentromeric ITSs (*open arrowheads*) in the same subset of chromosomes (**d**). Remaining ITSs (*open arrowheads*) are confined to a 45S rDNA region on chromosome pair 8 (compare pics. **b** and **d**) and to p-arms of st-a chromosomes. Finally, compare chromosome pair 8 on pics. **b**, **c** and **d**; entire q-arms bearing 45S rDNA/ITS are weakly C-positive after C-banding procedure. Bar = 10 μm

**Fig. 5:**
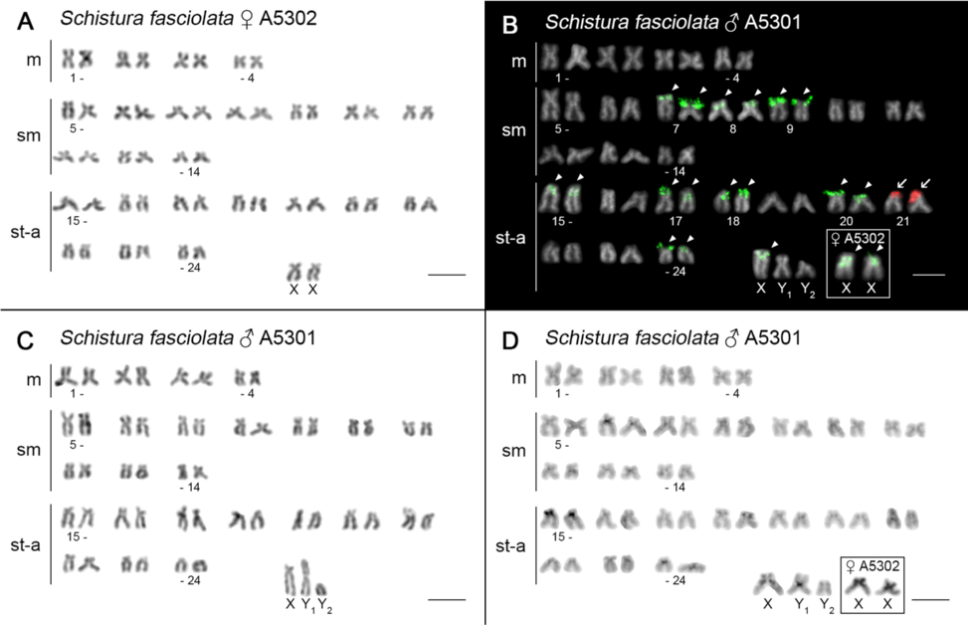
Karyotypes of male and female of *S. fasciolata* after different cytogenetic protocols. **a** female (2n = 50), **b**-**d** male (2n = 51). **a**, **c** conventional Giemsa staining, **b** dual-colour FISH with 45S rDNA (*red, arrows*) and 5S rDNA (*green, arrowheads*) probes, **d** C-banding. Putative sex chromosomes of female are boxed **b**, **d** next to those of the male karyotype. Note the presence of 5S rDNA site (**b**) and prominent pericentromeric heterochromatic region (**d**) on a putative X chromosome. Notice also a centromeric C-band on Y_1_ (**d**). Bar = 10 μm

Figure 2 summarizes 2n, karyotype structure, NF and rDNA phenotypes (i.e., number and position of both major and minor rDNA sites) within the phylogenetic tree context analysis. Seventeen out of 19 species displayed karyotypes with uniform 2n = 50, but with a marked variability in NF values (68–90) (Figs. 2, 3 and 5; Additional file 7: Figure S3). In the remaining two species, karyotypes with reduced 2n were observed: *N. ruppelli* (2n = 38) (Fig. 4a), *S. notostigma* (2n = 44 or 48) (Additional file 8: Figure S4A, C, E). Two different karyomorphs occured in examined individuals of the latter species – with 2n = 44 (five individuals, Additional file 8: Figure S4A, C) and with 2n = 48 (a single individual, Additional file 8: Figure S4E). Karyotypes of both species exhibited a significantly higher number of large m chromosomes compared to karyotypes with 2n = 50. Except for the large m chromosomes in *N. ruppelli* (six pairs) and *S. notostigma* (one or two pairs), karyotypes in all other species were composed of comparatively small chromosomes, gradually decreasing in size. Very tiny chromosomes were observed in *L. costata*, *P. pictilis*, *P. zonalternans*, *S. hypsiura* and *S. savona.* Centromere positions often gradually differed making it difficult to establish strict borderlines between formal chromosomal categories.

In almost all species no intraspecific numerical or structural polymorphisms between males and females that might indicate the presence of sex chromosomes were detected, although only females were examined in *L. costata* and *P. pictilis* and unsexed specimens in 7 other species – see Table 1. However, in *S. fasciolata*, males exhibited 2n = 51 chromosomes with a karyotype composed of (9 m + 20sm + 22st-a) while a female presented a 2n = 50 (8 m + 20sm + 22st-a), suggesting the presence of a multiple XY_1_Y_2_ sex chromosome system (Fig. 5a, c).

### Heterochromatin distribution and composition

The distribution of constitutive heterochromatin was studied by CDD (CMA_3_/DAPI) banding in all species and C-banding in a subset of 10 species (Additional file 9: Table S4). The C-banding and DAPI patterns were usually congruent with the exceptions observed in *N. ruppelli* and *S. bolavenensis*, where also some CMA_3_-positive (CMA_3_^+^) regions (NOR-associated) were slightly positively heteropycnotic after C-banding at the same time (Fig. 4c; Additional file 2: Figure S1L). In the remaining species, the CMA_3_^+^ regions did not match the C-bands. With the exception of *N. ruppelli* and *S. lendlii* (Fig. 4c; Additional file 2: Figure S1Q) all other species displayed generally low or moderate levels of AT-rich C-heterochromatin. In almost all species, its predominant location was in the pericentromeric regions of some or all chromosomes, except for *S. corica*, where only a few interstitial bands and two whole-arm heterochromatic regions (p-arms, sm) were apparent (Additional file 2: Figure S1M). In one or more chromosomal pairs of m-sm type in *M. guentheri, P.* sp., *S. bolavenensis*, *S. hypsiura* and *S. lendlii* (Additional file 2: Figure S1D, I, L, N, Q) the heterochromatin encompasses a substantial part or even the entire arm of the chromosome. These regions were adjacent to 5S or 45S rDNA only in *S. bolavenensis*, *S. corica* and *S. lendlii*. Few heterochromatic p-arms of st-a chromosomes were observed in *L. costata, M. guentheri, N. ruppelli*, *P. zonalternans* and *S. pridii* (Fig. 4c, Additional file 2: Figure S1B, C, D, G, O). Huge heterochromatic regions were found flanking the primary constrictions of m chromosomes in *N. ruppelli* (six pairs, Fig. 4c), *S. notostigma* (one or two pairs, Additional file 8: Figure S4C), *S. savona* (one pair, Additional file 2: Figure S1P) as well as in one (male) or two (female) st chromosomes in *S. fasciolata* (Fig. 5d). In the latter species, compared to Giemsa-stained karyotypes, this heterochromatic region was confined to the st chromosome present on one homologue in males and on both homologues in females. Also noticeable was the C-heterochromatic block on the male-specific single large m chromosome. Furthermore, intercalar DAPI-positive bands were clearly visible after C- or CDD banding in a subset of sm/st chromosomes (from one to four pairs) in *B. barbatula*, *N. ruppelli*, *N. binotatus, S. bolavenensis*, *S. corica* and *S. notostigma*, often appearing as dot-like sites located proximally on the q arms. Finally, a polymorphic AT-rich p-arm was observed in one homologue of pair 18 in *M. guentheri,* but only in the male karyotype (Additional file 7: Figure S3D).

**Fig. 6:**
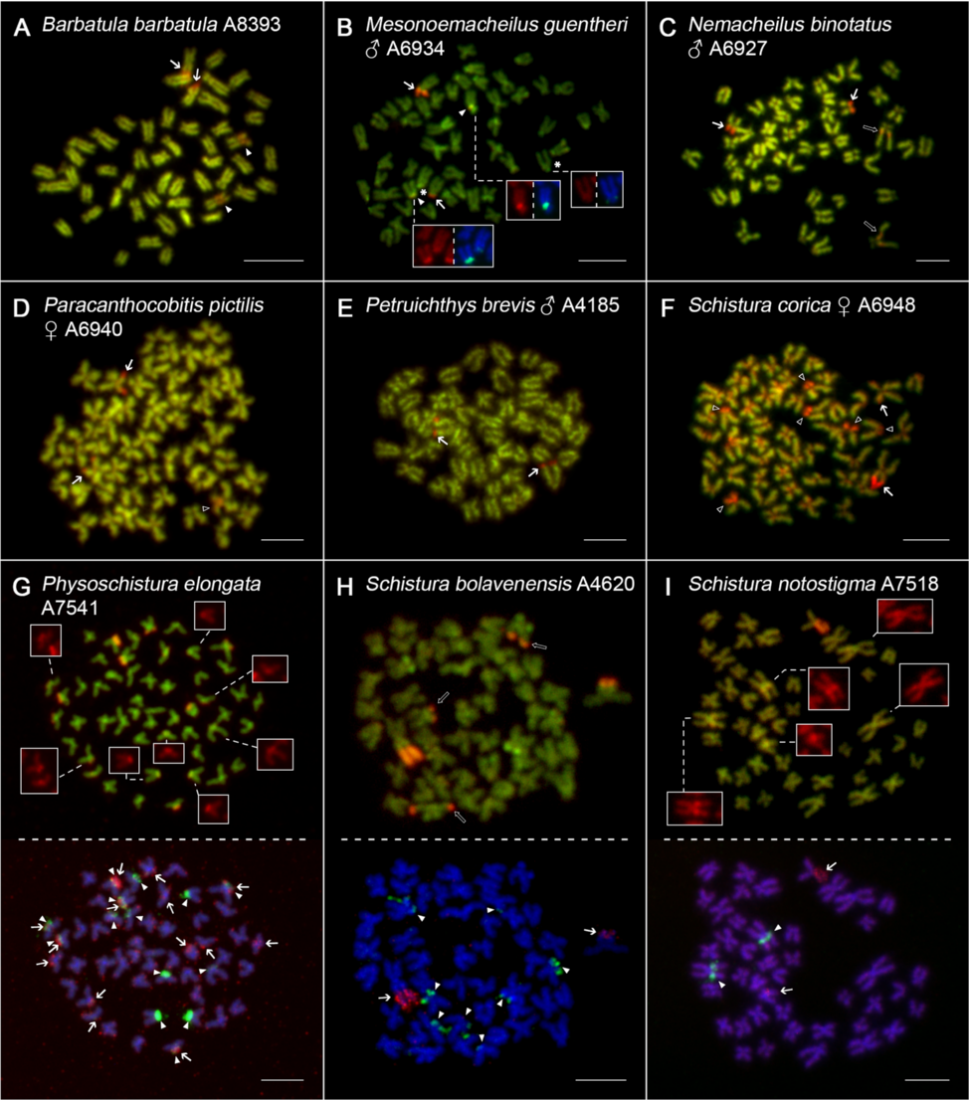
Mitotic metaphases of selected nemacheilid species after CDD banding. **a**, **c**, **d**, **e**, **f** single metaphases; **b** metaphase arranged with boxes showing particular chromosomes sequentially after CDD banding and dual-colour rDNA FISH. **g**-**i** whole metaphases arranged sequentially – after CDD banding (*upper row*) and corresponding dual-colour FISH showing locations of 45S rDNA and 5S rDNA (*lower row*). **a** *B. barbatula*, **b** *M. guentheri*, **c** *N. binotatus*, **d** *P. pictilis*, **e** *P. brevis*, **f** *S. corica*, **g** *P. elongata*, **h** *S. bolavenensis*, **i** *S. notostigma*. For better contrast, CDD-banded pictures were pseudocoloured in red (for CMA_3_) and green (for DAPI). FISH metaphases follow the same colour scheme as in Figs. 2, 3, 4 and 5. Arrows show CMA_3_^+^/45S rDNA sites, arrowheads show CMA_3_^+^/5S sites, open arrowheads show a putative CMA_3_^+^/5S sites and open arrows show CMA_3_^+^ regions non-related to rDNAs and minor/putative CMA_3_^+^ sites. In the particular case of *M. guentheri* (**b**), note the CMA_3_-negative 5S rDNA sites (*denoted by asterisk*), while the remaining boxes clearly show CMA_3_^+^/5S rDNA sites. In non-sequential metaphases (**a**-**f**), considering the number and location of CMA_3_^+^ signals in comparison to respective FISH karyotypes (Fig. 2 and Additional file 7: Figure S3), the association between 45S rDNA and CMA_3_^+^ sites is clearly apparent from pics. and the same is true also for some or all 5S rDNA sites in (**a**, **d** and **f**). Due to the close proximity of 5S rDNA sites to centromeres (which are usually AT-rich and display bright fluorescence), some CMA_3_^+^/5S rDNA sites are not clearly apparent from the pictures, therefore they are boxed with a separate channel for CMA_3_ (*red*) (**b**, **g**, **i**). Note the significant spreading of CMA_3_^+^ regions in centromeres of *S. corica* (**f**) and CMA_3_-positive ITSs in *N. binotatus* (**c**). Bar = 10 μm

CMA_3_ labelled only GC-rich regions associated exclusively with NORs in seven species (*L. costata*, *P. brevis*, *P.* sp., *S. fasciolata*, *S. pridii*, *S. savona* and *S. lendlii*)*,* but also with 5S rDNA regions in seven other nemacheilids (Additional file 9: Table S4). More specifically, species with only some 5S rDNA sites being CMA_3_^+^ (e.g., *M. guentheri*, *P. zonalternans*, *P. elongata*) (Fig. 6b, g; Additional file 10: Figure S5B) and others with all of them (e.g., *B. barbatula*, *N. ruppelli*, *S. notostigma*) (Figs. 4b and 6a, i). In six out of seven species, we observed the 5S rDNA/CMA_3_^+^ pattern directly by sequential application of CDD banding and rDNA FISH (Fig. 6b, g-i) In *S. corica* (Fig. 6f), however, a similar conclusion was based on observation of remarkably high number of CMA_3_^+^ sites and their distribution in centromeres and chromosomal p-arms, similarly to 5S rDNA sites. In *P. pictilis*, *P. lucidorsum* and *S. hypsiura*, association of CMA_3_^+^ and 5S rDNA sites was inconclusive (Fig. 6d; Additional file 10: Figure S5D, F). In *N. binotatus* and *S. bolavenensis*, CMA_3_ labelled NORs and some other regions non-related to 5S rDNA (Fig. 6c, h). A more complicated pattern was observed in *P. elongata* (with a subset of CMA_3_^+^ 5S rDNAs and additional CMA_3_^+^ regions) and *S. notostigma* (where all 5S rDNAs were CMA_3_^+^ and other CMA_3_^+^ regions also appeared) (Fig. 6g, i; Additional file 10: Figure S5G). Finally, *S. corica* displayed an extensive dispersal of CMA_3_^+^ regions with locations in all centromeres, some p-arms and along the single pair of NOR (Fig. 6f).

### rDNA phenotypes

All karyotypes resulting from the rDNA FISH experiments are shown in Figs. 3, 4 and 5; Additional file 7: Figure S3, Additional file 8: Figure S4B, D, F and Additional file 11: Figure S6B, D and partial idiograms showing rDNA phenotypes in the phylogenetic context are summarized in Fig. 2. In most species, the 28S rDNA probe (i.e., corresponding to the NOR-associated major ribosomal cluster 45S rDNA, which codes for 28S, 5,8S and 18S rRNA genes) showed only one pair of NOR-bearing chromosomes located in CMA_3_^+^ sites. NOR phenotypes with two or more loci were observed in *P. zonalternans* (Additional file 11: Figure S6B), *S. savona* (not shown; see later in the text), *S. lendlii* (Additional file 7: Figure S3V) and especially in *P. elongata* with the number of sites ranging from 12 (Additional file 11: Figure S6D) to 14 (Fig. 6g). In the latter, not more than six NORs were stained also by AgNO_3_ impregnation (data not shown). The 45S rDNA sites were located exclusively terminally (m) or covered entire p-arms of a particular st-a chromosome pair. By contrast, we found a considerable variability in the number of 5S rDNA sites, ranging from two (*B. barbatula*, *N. binotatus*, *N. ruppelli*, *S. notostigma*; Figs. 3b and 4b; Additional file 7: Figure S3F and Additional file 8: Figure S4B, D) to 20 (*Physoschistura* sp.; Fig. 3d). The 5S rDNA clusters were mainly located in pericentromeric regions or distributed in the entire p-arms of some st-a chromosomes, but location in/nearby centromeres of m-sm chromosomes was also observed (*B. barbatula*, *N. ruppelli*, *P. lucidorsum*, *S. notostigma*; Figs. 3b and 4b; Additional file 7: Figure S3L and Additional file 8: Figure S4B, D). In two species we observed one pair of chromosomes with a syntenic association of both rDNA classes (*P. brevis* – pair 13, Additional file 7: Figure S3J; *N. ruppelli* – pair 8, Fig. 4b) and another two species displayed direct co-localization of them (*P. zonalternans* – pair 12; *P. elongata* – pairs 4 and 12 – Additional file 11: Figure S6B, D). In the latter species there is an intraspecific variability in the number of both rDNA clusters as well as the number of their co-localization sites, based on observation of 5S rDNA ranging between 14 and 16 sites (Fig. 6g and Additional file 11: Figure S6D) and even six co-localized rDNA sites in some metaphases (Fig. 6g). Here, we further observed intraspecific variability in 1) size polymorphism, especially in 45S rDNA (best seen on FISH karyotypes of *S. bolavenensis* and *S. corica –* Additional file 7: Figure S3N, P) 2) polymorphism in the presence/absence of homologous rDNA sites (*P. pictilis* – pair 10; *S. lendlii* – pair 3 and *S. notostigma –* pairs 12 and 22, Additional file 7: Figure S3H, V, Additional file 8: Figure S4D, F), 3) number of rDNA sites (*S. corica* - pair 17; *S. hypsiura* – pair 18, Additional file 7: Figure S3P, R; *S. notostigma* - compare Additional file 8: Figure S4B and F), 4) heterozygosity for inversion involving rDNA loci (*P. zonalternans* – pair 10; *P. elongata* – pair 5, Additional file 11: Figure S6B, D), and 5) linkage of the 5S rDNA locus to a putative sex chromosome (*S. fasciolata –* Fig. 5b). Interestingly, a conspicuous difference in the 5S rDNA phenotype was discovered between two karyomorphs of *S. notostigma*. While the karyomorph with 2n = 48 (1 specimen) exhibited five sites of 5S rDNA (all in pericentromeric regions of the st-a chromosomes, Additional file 8: Figure S4F), the karyomorph with 2n = 44 displayed only two of them, adjacent to centromeres of large-sized m chromosomes (5 specimens) (Additional file 8: Figure S4B, D). In *S. savona*, we observed considerable intraspecific variability being shown from two to four signals of 45S and from two to eight signals of 5S rDNA cluster (data not shown). In this species, however, it was not possible to conclusively distinguish whether this was the result of high intraspecific and intra-individual variability or whether it was artificial due to the limited visibility of the hybridization signals on such extraordinarily small chromosomes and so, these results are not discussed further.

### Telomeric FISH

**Fig. 7:**
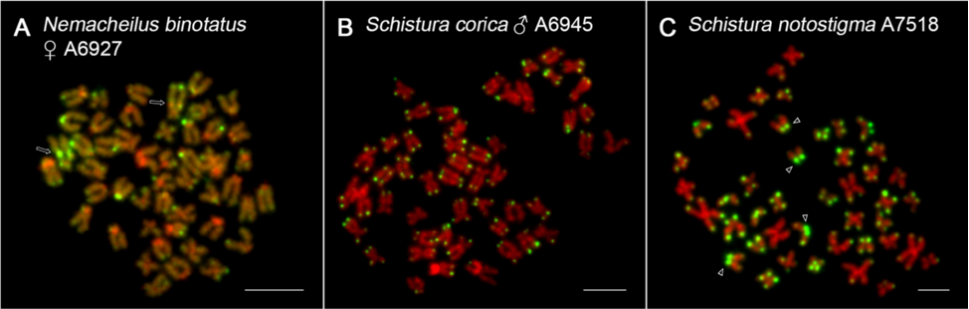
Mitotic metaphases of selected nemacheilid species after TSA FISH with telomeric (TTAGGG)_n_ probe. **a** *N. binotatus*, **b** *S. corica*, **c** *S. notostigma* (karyomorph with 44 chromosomes). Chromosomes with the telomeric repeat probe (*green colour*) are counterstained with DAPI, pseudocoloured in red colour for better contrast. Arrows point to the chromosomes with ITSs (**a**). Arrowheads show telomeric probe covering entire p-arm of st chromosomes (**c**). Bar = 10 μm

In order to document interstitial telomeric sites (ITSs) as remnants of chromosomal rearrangements, we employed FISH with conserved vertebrate telomeric (TTAGGG)_n_ repeat [44] in a subset of seven species (*L. costata*, *N. binotatus*, *N. ruppelli*, *P. brevis*, *P. elongata*, *S. corica* and both karyomorphs of *S. notostigma*). As expected, the telomeric probe labelled the ends of all chromosomes, and no ITSs were revealed in five out of seven species (Fig. 7b, c and Additional file 12: Figure S7A-D). Clear ITSs, however, were observed consistently on ten metaphases of *N. binotatus* (Fig. 7a) and 15 metaphases of *N. ruppelli* (Fig. 4d). In *N. binotatus*, a single pair of ITSs occurred proximally on the q-arms of the largest chromosome in the karyotype (pair 11). These ITSs co-localized with sequentially heterogeneous AT/GC-rich heterochromatic regions. In *N. ruppelli*, three pairs of extensive and three pairs of faint pericentromeric ITSs were observed in large-sized m chromosomes (Fig. 4d). These six ITSs were coincident with AT-rich C-heterochromatin (Fig. 4c). Moreover, in this species additional large ITSs were also scattered all along the region of the single pair of 45S rDNA (compare Fig. 4b and d). The high intensity of some ITSs signals resulted in very limited visibility of natural telomeric signals on the chromosomal ends. Finally, in *N. ruppelli* and *S. notostigma*, some p-arms of small or medium-sized st-a were entirely covered by telomeric repeats.

## Discussion

### Topology of the phylogenetic tree

In our phylogenetic reconstruction the seven analysed species of *Schistura* do not form a monophyletic lineage, but appear as polyphyletic. This result reflects the massive flaws in the present taxonomy of this genus as already formerly stated by several taxonomists who referred to *Schistura* as ‘a provisional, polyphyletic assemblage’ [45], ‘polyphyletic’ and ‘waste-basket name’ [46] or ‘possibly not monophyletic’ [47]. The observed polyphyly of *Schistura* is therefore not surprising, but most likely reveals the true natural relationships between the analysed taxa. The two analysed species of *Physoschistura* turn out not to be closely related, supporting the former opinion that *P. elongata* is not closely related to the Burmese species of *Physoschistura* [47].

### Karyotype differentiation and evolution

Karyotypes of *B. barbatula*, *L. costata*, *P. pictilis*, *S. fasciolata* and *S. savona* were revisited, whereas the remaining 14 species were examined for the first time. Our study thus increased the number of karyologically described river loaches to 38. Comparison of nemacheilid karyotypes reported in former studies with results presented here (Fig. 2) showed a different degree of congruence. While in *B. barbatula* our karyotype description matched the previous report of Vasil’ev [48] only, the karyotype of *P. pictilis* (formerly as *Acanthocobitis botia*) differed slightly in morphological classification from that recorded by Rishi et al. [49]. Also, we evaluated the karyotype of *L. costata* as having a higher number of biarmed elements than in Kim et al. [50]. Moreover, the karyotype of *S. fasciolata* described here is not consistent with that reported by Yu et al. [19], where no sex chromosomes were found, but one specimen of *S. fasciolata* with 44 chromosomes was included. Finally, karyotypes of *S. savona* reported in Khuda-Bukhsh et al. [18] consisted of 36 chromosomes while our results showed uniformly 2n = 50 in all three examined specimens. All these discrepancies may have resulted - besides the differences in chromosomal morphology classification due to difficulties described in the previous section – from the description of chromosomally different populations or by the misidentification of some species in the earlier studies.

Seventeen out of 19 analysed species showed conserved karyotypes with 2n = 50 (Figs. 2, 3 and 5; Additional file 7: Figure S3 and Additional file 11: Figure S6). This 2n has been already documented for the majority of previously surveyed river loaches [7, 12–14] as well as in some other loach families [4] and cyprinid fishes [7, 51]. Similar karyotypes (with either 48 or 50 chromosomes) were found in more than 50 % teleost species, thus indicating high conservativeness of this 2n [52]. Additionally, the 2n = 48 with exclusively monoarmed chromosomes is hypothesized to be ancestral for all Teleostei [53, 54].

Despite the generally stable karyotype macrostructure, the river loaches analysed here varied greatly in the proportion of chromosome types reflected by the increase or decrease of the NF value. The occurrence of species with similar karyotypes did not correspond with their phylogenetic relationships. Changes of NF without changes of the 2n are strong indicatives that nemacheilid chromosomes have evolved by diverse intrachromosomal rearrangements, such as various types of centromeric shifts.

We further recorded two species with karyotypes exhibiting reduced 2n, namely *N. ruppelli* (2n = 38) and *S. notostigma* (2n = 44 or 48). In the latter, our sample included two different karyomorphs. A single individual with 48 chromosomes did not show any significant differences in morphology and in sequences of *IRBP*, *RAG1* and *cyt b* in comparison to individuals with 2n = 44. Since we do not know the exact localities of analysed specimens, we cannot conclude, whether this result indicate the interpopulational variability.

In both species, reduction in 2n was accompanied by an increased number of large m chromosomes, implying their origin via one or several centric fusions of Robertsonian (Rb) type. Based on comparison of 2n and NF [55] and with respect to prevailing 2n = 50 in examined nemacheilids, *N. ruppelli* most likely underwent six Rb translocations, while karyotype differentiation in *S. notostigma* probably involved one Rb translocation (in karyomorph 2n = 48) and two Rb translocations, one tandem fusion and one para/pericentric inversion (in karyomorph 2n = 44), respectively. According to our phylogenetic analysis (Fig. 2), *N. ruppelli* and *S. notostigma* are not closely related, therefore the reduction of 2n in these species apparently represents independent events. Furthermore, the combined results from C-banding and telomeric FISH suggest a slightly different scenario of karyotype changes in both species (see below).

Besides our study, the evidence of reduced 2n among Nemacheilidae has already been documented for *Nemacheilus selangoricus* (2n = 40) [17], *Paracobitis potanini* (2n = 48) [19], *S. fasciolata* (2n = 44) [19], *S. savona* (2n = 36) [18] and *Triplophysa siluroides* (2n = 48) [20]. A different bias towards an increased number of either mono- or biarmed elements was apparent in these species. Some nemacheilid species (or at least representatives from some subpopulations) thus tend to reduce their 2n via centric or tandem fusions. Except for the studied males of *S. fasciolata* here and the report on triploidy [21], karyotypes of river loaches analysed to date did not exceed 2n = 50 ([7, 12–14], this study).

Our data show that karyotypes in nemacheilid loaches have diversified mainly via centric or tandem fusions and pericentric inversions. In general, such chromosomal rearrangements can act as an efficient barrier for gene flow (by suppressing recombination in the affected region) and thus can contribute to speciation and/or local adaptation processes [56–58].

### Distribution and sequence composition of constitutive heterochromatin

Heterochromatin is an important source of karyotype diversification in several fish groups (e.g., [27]) and its unusual distribution may sometimes correspond to remnants of particular chromosome rearrangements [59]. As we present here, the karyotypes of river loaches differ greatly in their distribution of AT-rich C-heterochromatin (Additional file 9: Table S4), and contain some noticeable common patterns. We especially emphasise a) the dot-like intercalary heterochromatic bands on the q-arms of sm or st chromosomes, very close to the centromere (e.g., in *B. barbatula*, *N. binotatus*, *S. bolavenensis* and *S. notostigma*) and b) the presence of entirely heterochromatic arms in some elements (e.g., in *M. guentheri*, *S. bolavenensis*, *S. corica*, *S. hypsiura* and *S. lendlii*). Both observed patterns might be related to pericentric inversions (heterochromatinization of short arms are usually the result of this kind of rearrangement) and/or heterochromatin block addition [60]. The dot-like intercalary sites could be also explained by tandem fusions [60, 61], but considering the constant 2n in the majority of species under study, it would only be a plausible explanation for *S. notostigma*. Interestingly, although the presence of large biarmed chromosomes with entirely (or almost entirely) heterochromatic arms was shared by five nemacheilid species (*M. guentheri*, *P*. sp., *S. hypsiura*, *S. corica* and *S. lendlii*), these regions were adjacent to rDNA clusters only in two of them (*S. corica* and *S. lendlii*). Whether these chromosomes are homeologous among some of the mentioned species and whether the heterochromatic blocks contribute to the dynamics of rDNA clusters remains inconclusive. Also, polymorphism regarding the addition of AT-rich heterochromatic p-arms was observed in one pair of chromosomes in *M. guentheri*. While the male was heterozygous for the presence of a prolonged heterochromatic arm, the female possessed only the short variants. Comparable results have been previously documented in some other fishes ([62] and references therein) and may be explained by an unequal crossing-over or by transposition/amplificaton processes involving a DAPI-rich centromeric region [22]. The last intriguing feature was the presence of large blocks of AT-rich heterochromatin in the pericentromeric region of the largest m chromosomes of *N. ruppelli* and *S. notostigma*. These regions are possibly remnants of pericentromeric heterochromatin of previously monoarmed elements. Similar feature displayed also *S. savona* on one m chromosome pair, however, with unreduced 2n = 50.

In fishes, GC-rich DNA segments labelled by CMA_3_ are almost exclusively associated with NORs [63, 64], with some exceptions in sturgeons [65]. NOR regions were usually not visualized after C-banding, thus most likely suggesting that GC-rich sequences were inserted into the intergenic spacers (IGSs) of the 45S rDNA arrays [63, 66]. Additionally, nearly half of the species analysed showed further CMA_3_^+^ sites restricted to 5S rDNA regions – a feature that up to now has only been found among fishes in some Polypteriformes [67] and Perciformes, namely in Centrarchidae [68], Pomacanthidae [69] and Gobiidae [70]. Deiana et al. [68] attributed this feature to the presence of GC-rich repeats in NTS. Particularly interesting was the observation of all centromeres being CMA_3_^+^ in *S. corica* – a similar feature as, for instance, in Gobiidae [71, 72] and Polypteriformes [73]. Also in the genus *Cobitis*, high number of CMA_3_^+^ regions were recorded which were non-related to NORs (together with CMA_3_-negative NOR sites) [74]. In a recent study, some CMA_3_^+^ regions non-related to NORs were observed also in *P. elongata*, *S. bolavenensis* and *S. notostigma*. Therefore, our results represent another example that CMA_3_-staining and 45S rDNA FISH do not always correspond and that CDD banding itself is not sufficient for the proper identification of NORs in fishes (discussed in [75, 76]).

The scattered occurrence of non-45S rDNA GC-rich sites does not appear to imply any correlation with phylogenetic relationships. However, the phylogenetically most derived species (*P. brevis* and *Physoschistura sp.*) apparently lack GC-rich 5S signals (Fig. 6e and Additional file 10: Figure S5C). The evolutionary significance of this type of variability is still under debate. For instance, the sequence composition of heterochromatin can be associated with the different success of recombination processes and with a propensity to some kind of chromosome rearrangements. Here, the GC-rich regions were involved in two Rb translocations in *S. notostigma* and one of the resulting fusion points also involved a 5S rDNA site, because karyomorph with 2n = 48 display higher number of exclusively terminally located 5S rDNA and GC-rich sites, while karyomorph with 2n = 44 exhibit reduced number of such regions, with some of them being apparently re-located to the pericentromeric region of large m chromosomes (Fig. 6i; Additional file 8: Figure S4B, F and Additional file 10: Figure S5G). Hence, centric fusion is very likely partly responsible for the reduction of 5S rDNA sites from five (karyomorph with 2n = 48, st chromosomes, Additional file 8: Figure S4F) to two (karyomorph with 2n = 44, m chromosomes, Additional file 8: Figure S4B, D). A similar scenario could also explain the largest sm pair in *N. ruppelli* (no. 8, Fig. 4b), with GC-rich 5S rDNA in the centromeric region. However, considering the other six pairs of large m chromosomes with marked large pericentromeric heterochromatin and ITSs (as evidence of Rb translocation; Fig. 4c, d), there is no space for additional fusions since 2n = 38 had already been reached. Therefore, two alternative explanations for this discrepancy can be hypothesized: 1) the occurrence of conspicuous pair 8 in *N. ruppelli*, with syntenic association of both rDNAs, may be the result of Rb translocation only in the case of parallel fission of some other previously metacentric pair (resulting possibly in st-a pairs 18 and 19, with a markedly strong telomeric signal on the p-arms, Fig. 4d) or 2) synteny of both clusters on chromosome pair 8 has been caused by another type of translocation event, non-affecting the 2n.

GC-rich regions are more prone to high recombination rates [77]. In a similar way, GC-rich centromeres have been hypothesized to be favoured or even essential in the process of Rb translocations in some gobiid fishes [71, 72]. On the other hand, the majority of Rb translocations in *N. ruppelli* originated from elements containing AT-rich centromeres, and therefore it appears that more mechanisms exist for Rb translocations besides involvement of GC-rich regions. These findings contrast with those studies, but are consistent with results observed in killifishes [22] and *Mus musculus domesticus* [78].

Due to the number of reports evidencing 5S rDNA in the centromeres of fused chromosomes are gradually increasing in fishes [79, 80], it raises the question whether the 5S rDNA region could contribute in some way to the fusion process or it is only a consequence of it. It has been suggested that 5S rDNA can serve as breakpoints for the fusion due to its intensive activity and chromatin decondensation [80] but further data supporting this hypothesis would be required.

In our study, GC-rich sequences may be involved in the dispersion and homogenization of GC-rich/5S rDNA sequences as well as 45S rDNA sites and 5S/45S co-localized sites in the genome of *P. elongata* by ectopic recombination, similarly as observed in Gobiidae [70]. However, other nemacheilid species bearing GC-rich/5S rDNA regions do not display such extensive dispersion of 5S rDNA. Thus, other factors such as transposition together with stochastic processes in isolated populations may have been involved in the dynamics of GC-rich/5S rDNA sites. Similarly, a combination of transposition and unequal crossing-overs could have contributed to the dispersion of GC-rich centromeres in *S. corica*.

Our results from C- and CDD- banding further reinforced our initial hypotheses about the roles of pericentric inversions and centric/tandem fusions as the main processes underlaying the karyotype differentiation of examined river loaches. Collectively, our data point to a substantial heterogeneity both in heterochromatin distribution and composition among the analysed river loaches, resulting probably from intense dynamics at chromosomal and genomic levels.

### Sex chromosomes

While the majority of analysed species lacked morphologically differentiated gonosomes, we identified a putative multiple sex chromosome system XY_1_Y_2_ in *S. fasciolata*. The two Y chromosomes in males (m and st) possibly arose from a double-strand break (or fission) in one proto-Y chromosome, followed perhaps by intrachromosomal rearrangements, such as pericentric inversions, in the larger element. Interestingly, the FISH results showed a pericentromeric 5S rDNA site on a putative X chromosome – a situation previously observed e.g., in rainbow trout [81]. In general, about 10 % of fish species cytogenetically examined to date exhibit morphologically differentiated gonosomes [82] and within them, only a few cases of the multiple system XY_1_Y_2_ have been reported (e.g., [83–85]), with apparently phylogenetically independent origins among genera and families. Our finding is the first observed in river loaches. However, because our sample was rather small, we can not exclude the possibility that we are still dealing with a polymorphism instead of a sex chromosome system. Therefore our conclusions should be further confirmed using comparative genomic hybridization (CGH) [86] and analyses of meiotic chromosomes on a larger sample base.

### Telomeric FISH pattern

Tandemly-arrayed telomeric (TTAGGG)_n_ repeats are usually present at the ends of vertebrate chromosomes, ensuring their stability and integrity. However, they also occasionally appear in non-telomeric locations (ITSs), possibly as putative markers of previous chromosomal rearrangements, transpositions or as the result of DNA repair mechanisms ([87–89] and references therein). In *L. costata, P. brevis, P. lucidorsum* and *S. corica* and both karyomorphs of *S. notostigma*, the telomeric signals were restricted to the chromosome ends. Although some metaphases displayed putative intercalar telomeric sites, the lack of a second terminal signal on the particular chromosome suggests that these signals label natural telomeres. ITSs were therefore only found in *N. ruppelli* and *N. binotatus*. In the latter species, the single prominent ITS located interstitially on the long arm of the largest st pair may indicate a pericentric inversion or a tandem fusion event. Since the ancestral diploid chromosome number (2n = 50) remained unchanged, the observed pair of ITSs is rather a relic of a previous pericentric inversion, although such types of rearrangement are not frequently associated with retained telomeric repeats in vertebrates ([90, 91]). The intense telomeric signal may be the result of an additional amplification of telomeric repeats either before or after the rearrangement, or, in the case of *N. binotatus,* could have originated from a relatively recent pericentric inversion. The failure to detect ITSs in the majority of the remaining species does not necessarily mean, that inversions did not occur as it is possible that the residual traces of ITSs have been lost or reduced to such a low copy number as to be undetectable by FISH analyses. The telomeric FISH also provided the interesting evidence that the mechanism of Rb translocations differs significantly between species with reduced 2n (*N. ruppelli* and *S. notostigma*). While *N. ruppelli* possessed several huge pericentromeric ITSs, none were found in *S. notostigma*. As described by Slijepcevic [92], the mechanism of Rb translocations can be either 1) associated with a loss of telomeric sequences prior to fusion or 2) with their preservation in otherwise inactivated telomeres. Moreover, there is also the possibility that 3) some degenerate telomere-like sequences may become part of the centromeric heterochromatin and subsequently expand along this region as a result of the action of a variety of amplification mechanisms [92, 93]. We suggest that a combination of scenarios 2) and 3) apply in *N. ruppelli*, while *S. notostigma* followed the first scenario, hence residual telomeric sequences were absent at the fusion points. The assumption of amplified centromeric ITSs in *N. ruppelli* is based on their remarkably stronger signal compared to native telomeres and on their C-positive character. ITSs often co-localize with heterochromatin blocks [87, 88, 94] and large, mostly centromeric ITSs similar to those in *N. ruppelli* (sometimes referred to as heterochromatic ITSs, or “het-ITSs”), have previously been described in other fishes as well as in a variety of other organisms [88, 89]. Interestingly, additional ITSs were found to be associated with the 45S rDNA cluster in *N. ruppelli* as confirmed by FISH and CDD banding. This association has been previously described in Anguilliformes, Mugiliformes, Salmonifromes and Syngnathiformes [89] and was believed to play a role in the silencing of additional 45S rDNA copies [95]. This seems unlikely in *N. ruppelli*, however, as the telomeric repeats perfectly match with the entire region of the only pair of 45S rDNA. Alternatively, the mechanism of rDNA silencing could be more complex or prone to leakage of rDNA expression in some way. Finally, large ITS-like blocks covering entire p-arms of some monoarmed chromosomes as observed in *N. ruppelli* and *S. notostigma* bring another example of enormous nemacheilid cytogenetic variability.

### Genomic organization and distribution of rDNA clusters

Mapping of tandemly-arrayed repetitive sequences has proven to be an important tool for karyotype analysis [59] and this is especially true for ribosomal RNA genes. The rDNA phenotypes are often species-specific and have been used as cytotaxonomic markers [96]. However, a number of reports demonstrating extensive inter/intra-specific variability of these markers is still growing in fishes [97–100], other animal groups [101] and plants [102]. Here, we point to the conservative NOR phenotype, presented by one pair bearing 45S rDNA signals in 15 out of 19 nemacheilid species. Although the possibility of interspecific homeology of NOR-bearing chromosomes is rather unlikely, definitive proof based, for instance, on the approach described by Milhomem et al. [103] is missing. From all our samples we documented multiple 45S rDNA sites only in *P. elongata* (Fig. 2). Subsequent analysis made by silver staining detecting only NORs actively transcribed in the preceding metaphase [104] revealed not more than six loci (data not shown), thus, some extra loci are either nonfunctional or silenced.

We observed a conservative NOR phenotype of one major rDNA bearing pair – a pattern found in more than 70 % of examined fish species to date [76]. In contrast, we detected a considerable variability in the pattern of 5S rDNA ranging from two to 20 sites (Fig. 2). The presence of a single pair of both rDNA clusters is thought to be the plesiomorphic condition in teleost fishes, whereas two or more chromosome pairs bearing either 45S or 5S rDNA sites represent a derived condition [76, 105]. In our study, only *B. barbatula* and *N. binotatus* exhibited karyotypes with the ancestral 2n = 50 together with one pair of both rDNA clusters and in *B. barbatula*, our results confirmed the previously reported NOR phenotype based on silver staining [15]. In the remaining two species exhibiting the characteristic teleost rDNA phenotype the karyotypes were derived (*N. ruppelli* and *S. notostigma*). A variable 5S rDNA pattern in combination with a conservative NOR phenotype has been observed in some fish groups [70] while other fish groups have shown the opposite situation (variable 45S and conservative 5S rDNA; [106]).

The 45S rDNA site has a predominantly terminal position on the different chromosomes of the analysed species, while the 5S rDNA is located almost exclusively in the pericentromeric regions or it covers entire p-arms of monoarmed chromosomes. Pericentromeric or, more generally, interstitial position of 5S rDNA appears to be universal among fishes [107].

In fishes, the chromosome locations of both rDNA multigene families are usually on different chromosomes, perhaps due to 1) the elimination of possible rearrangements between both multigene families and 2) to allow rDNA clusters to evolve independently [105, 108]. On the other hand, exceptions with syntenic location or direct co-localization of both rDNA clusters (or their linkage to other multigene families) has already been documented in a variety of vertebrates [109, 110], including reports from several fish groups [111] as well as in loaches of the family Cobitidae [98], a sister lineage to nemacheilids. This pattern is rather patchily distributed across the phylogenetic trees and was also evidenced in our study. In *N. ruppelli*, the 5S rDNA loci occupied the pericentromeric region of a big m chromosome while the 45S rDNA was situated terminally on the q-arm of the same chromosome. In *P. brevis*, a similar association was observed in one pair of big st elements. Moreover, direct co-localization of rDNA clusters was observed in two species: one pair in *P. zonalternans* and from four to six co-localized sites in *P. elongata*. Such a rare situation has probably no evolutionary advantage as both classes of rRNA genes are transcribed by different RNA polymerases [109]. Therefore, this constitution is a possible consequence of recent genome instability and reshuffling as typically observed in hybridization events [100].

In all species analysed here, a size polymorphism in the homologous 45S rDNA sites was apparent. Such an observation is relatively common among fishes and is attributed to the processes of unequal crossing-over or the amplification of adjacent heterochromatin [112]. We also observed an intraspecific polymorphism in terms of the number of rDNA sites present (*S. corica, S. hypsiura, S. notostigma*) and a polymorphism in the presence/absence of rDNA site on one homologous chromosome in *S. notostigma* (5S rDNA and 45S rDNA), *S. lendlii* (female, 45S rDNA) as well as for both females of *P. pictilis* (5S rDNA)*.* Unfortunatelly, the limited number of specimens available in our sample is insufficient to conclusively determine either fixation or heterogeneity of this feature in the population. Similar heterozygous constitutions of rDNA FISH signals have been commonly observed in several species of fishes (e.g., [112, 113]) including some from Cobitidae [74, 97, 98]. The lack of signal on one of the homoloques may be a direct consequence of sequence elimination due to unequal crossing-overs, often related to a process of concerted evolution in tandemly-repeated genes [114] or by the activities of repetitive DNA such as TEs [59, 115]. Finally, we also observed the polymorphism caused by rDNA loci inversion in *P. zonalternans* and *P. elongata*. This feature, present in Cobitidae [98], suggests a strikingly similar dynamics of rDNA loci in these closely related loach families as well as another clue about the contribution of inversions to the karyotype differentiation of river loaches.

Our study has revealed an extensive dispersion of multiplied sites of 5S rDNA and also of 45S rDNA in nemacheilids. The dominance of the ancestral 2n = 50 karyotype in Nemacheilidae refutes chromosomal rearrangements as the trigger mechanism for this dispersion, but amplification and dispersion of 5S rDNA clusters may also be caused by transposition and unequal crossing-over or ectopic recombination between various tandemly-arrayed sequences in adjacent heterochromatin [102, 115, 116]. Thus, rDNA clusters themselves can provide a substrate for non-homologous recombination, thereby promoting chromosomal rearrangements [101]. A significant fraction of the rDNA units in animals are interrupted by TEs highly specialized for insertion into conserved sites within the rRNA genes [114, 117] and recent studies suggested that they might cause rDNA mobility [118–120]. Co-localization of non-LTR RTEs of the Rex family with rDNA followed by a subsequent expansion of rDNA sites have been uncovered by FISH analyses for 5S [94] and 45S rDNA [121, 122]. It is tempting to hypothesize that a similar mechanism could cause the amplification of 5S/45S rDNA in other fish species with documented extensive rDNA dispersion. In our study, we have found the non-LTR retrotransposon L1-2_DR element, from the Tx1 clade (L1 lineage) – inserted close to a coding region of 5S rDNA in *S. pridii*. This element has been previously described in zebrafish [123].

Since the karyotype of *S. pridii* exhibit a large number of 5S rDNA loci (18), the L1-2_DR may have been inserted into the NTS of both analysed 5S variants and subsequently retrotransposed to other chromosomal loci. RTEs of this L1 family preferentialy jump into AT-rich regions [123], therefore the AT-rich pericentromeric heterochromatin of *S. pridii* located adjacent to 5S rDNA could serve as a primary location for this mechanism. Alternatively and/or as secondary consequences, the L1-2_DR (or other TEs) could provide the substrate for non-homologous (ectopic) recombination between centromeres of several chromosomes in *S. pridii* yielding to a dispersion of 5S rDNA to other sites. Both hypotheses deserve further investigation regarding the localization of L1-2_DR elements on the chromosomes of *S. pridii* and also the investigation of possible rDNA/TEs association in other river loaches through FISH analysis. However, we can not rule out the hypothesis, that L1-2_DR elements are just following the spread of 5S rDNA and not driving it (for additional note, see Additional file 1: Supplementary Discussion).

The variation observed in the distribution of 5S rDNA sites implies a complex microevolutionary mechanism behind the organization of nemacheilid genomes. The final questions are: wheter or not a dispersion of 5S rDNA is only a byproduct of rapid genomic change, is there any possible contribution to the host genome worth maintaining such a high number of copies, or are the excessive copies most likely sentenced to pseudogenization and elimination? Could an extensively elevated number of rDNA loci somehow contribute to the speciation process? We are still far from understanding this but some indications have come from studies on 45S rDNA in notothenoid fishes [111] and humans [124]. According to Pisano and Ghigliotti [111] the differential pattern of the rDNA phenotype could have a possible adaptive significance in subzero temperatures. Furthermore, the study of Gibbons et al. [124] shows that 45S rDNA dosage is correlated with mitochondrial DNA abundance and with the expression of some chromatin modifiers thereby affecting mitochondrial-related processes and changes in global gene expression. However, whether a similar correlation is true also for 5S rDNA dosage remains an open question (but see [125]). Thus, such an explanation does not yet fit our hypotheses about the mechanisms behind nemacheilid radiation success, although, it does suggest a frame in which to evaluate the contribution of multiple 5S rDNA to adaptation and speciation processes.

Conclusively, our data suggest frequent changes of 5S rDNA phenotypes in contrast to the stable pattern of 45S rDNA. Extensive variability of 5S rDNA loci may be regarded as an indicator of significant intragenomic processes [115, 116] and thus can be viewed in the context of an incipient stage of speciation, where evolutionary changes driven by the dynamics of repetitive DNA are currently in action [59]. This process can be also related to extreme ecological conditions possibly resulting in (re)activation of TEs [122]. As documented in several animal and plant species, elevated activity of TEs may contribute to adaptation to a new environment [126, 127]. Furthermore, the processes of transposition and/or ectopic recombination were not likely restricted only to regions of 5S rDNA. Numerous studies have documented the involvement of TEs in chromosomal rearrangements [59, 115, 126, 128]. We therefore conjecture that TEs might also contribute to the dynamics of nemacheilid genomes in this way.

### Phylogenetic and ecological inferences

We have used a phylogenetic tree to show the relationships between the analysed individuals. When mapped on this tree, the observed cytogenetic characteristics did not reflect the phylogenetic pattern, suggesting that certain cytogenetic character stages, like a lowered number of chromosomes, did not occur in closely related, but in non-related species. Therefore the parallel occurrence of cytogenetic character stages in two species is not the result of a single evolutionary event, but of convergence or parallel evolution. Our study has revealed a high variability in cytogenetic characters with almost none of them producing a phylogenetic signal. Therefore, a vast number of independent events with no general direction must have happened to cause the observed cytogenetic variability. The frequent occurrence of independent cytogenetic changes as revealed by the phylogenetic reconstruction further emphasises the high mutational activity of the nemacheilid genome at the cytogenetic level.

In contrast to the general observation of independent cytogenetic events, one of the variable cytogenetic characters did show an interesting pattern. The highest numbers of 5S rDNA loci (up to 20 sites) were almost exclusively observed in local endemics or inhabitants of small, fragmented habitats (*P. brevis*, *P. elongata, P.* sp., *S. hypsiura* and *S. pridii* – see Fig. 2). This produces a comparably small effective population size and therefore a small gene pool for the species, encouraging the establishment of new chromosomal patterns through genetic drift, meiotic drive and inbreeding [60]. In *P. brevis* the actual population size is quite large, but as it occurs only in a single lake, it can be assumed that the species has undergone through a serious bottleneck during the colonization of this area.

## Conclusions

Our data provides important information regarding the karyotype differentiation trends in Nemacheilidae. The majority of surveyed species showed the karyotype characteristics common for teleost fishes – e.g., 2n = 50 chromosomes with a slightly changing centromere position, a single pair of NOR and its association with GC-rich blocks of heterochromatin. However, a number of deviations were also apparent – e.g., reduced 2n in two species, atypical locations of GC-rich heterochromatin (e.g., in 5S rDNA sites), cases of multiple rDNA sites and the presence of putative sex chromosomes. While conventional staining showed prevailing uniformity of the nemacheilid karyotypical macrostructure, analysis at the molecular-cytogenetic level revealed much more variability and greater diversity than previously expected. An increased number of 5S rDNA sites were observed, especially in species with a small effective population size. The mechanisms responsible for such intense dynamics can possibly be attributed to the presence of repetitive sequences and could contribute to enormous success of Nemacheilidae in their colonization and exploitation of new niches, as well as with their adaptation processes. Our study presents river loaches as a new attractive model fish group for investigating the dynamics of cytogenetic markers in association with evolutionary and ecological questions. Importantly, we have also introduced a new non-invasive technique for obtaining chromosome spreads for molecular-cytogenetics protocols.

## Availability of supporting data

All the supporting data are included as additional files.

## Additional files

Additional file 1:
**Supplementary Methods 1.** Taxonomic identification of nemacheilids. **Supplementary Methods 2.** Preparation of chromosomes from regenerating fin tissue. **Supplementary Methods 3.** PCR conditions of 5S and 45S rDNA amplification. **Supplementary Methods 4.** PCR conditions of *RAG1*, *IRBP* and *cyt b* amplification. **Supplementary Discussion**. Possible functional consequences of excessive 5S rDNA copies. (PDF 181 kb) Additional file 2: Figure S1. Mitotic metaphases of selected nemacheilid species after C-banding or DAPI-staining. (A,D,C,F.G,H,I,J,M,O,P,Q) DAPI staining; (D,E,K,L,N) C-banding improved with DAPI counterstaining. Metaphases from both methods are converted to inverted pictures. (A) *B. barbatula*, (B) *L. costata,* (C,D) *M. guentheri* (E) *N. binotatus*, (F) *P. pictilis*, (G) *P. zonalternans*, (H) *P. brevis*, (I) *P.* sp., (J) *P. elongata,* (K) *P. lucidorsum*, (L) *S. bolavenensis*, (M) *S. corica*, (N) *S. hypsiura,* (O) *S. pridii*, (P) *S. savona,* (Q) *S. lendlii*. Arrows depicts whole-armed heterochromatin, arrowheads denote interstitial heterochromatin, the asterisk indicates C-positive NORs (as rare feature among species under study). For comparison of banding patterns between both methods, see pics. C and D. Note that several species share marked interstitial heterochromatic sites indicating the remnants of putative chromosomal rearrangements (e.g., pericentric inversion) (A,E,L,M,Q). Of particular interest are the completely heterochromatic arms in m-sm chromosomes occuring in a subset of species (C,D,L,M,P,Q). Note also heterochromaic p-arms in some st-a chromosomes (C,D,E,O,P). Bar = 10 μm. (ZIP 4276 kb) Additional file 3: Table S1. Primer sequences used in this study. (PDF 221 kb) Additional file 4: Table S2. GenBank accession numbers of *cyt b*, *IRBP* and *RAG1* sequences of nemacheilids and one botiid species (*B. lohachata*). (XLSX 17 kb) Additional file 5: Table S3. GenBank accession numbers of 5S and 28S rRNA sequences of four nemacheilids (*P. elongata, S. bolavenensis, S. corica, S. fasciolata*) and one botiid species (*B. almorhae*). (XLSX 14 kb) Additional file 6: Figure S2. Sequence alignment of cloned 5S rDNA fragments from *S. pridii.* Nucleotide sequences (5′-3′) obtained from both specimens (A7548, A7549) corresponding to the short (A) and long (B) variant of 5S rDNA, containing partial 5S rDNA coding sequence (green), partial sequence of L1-2_DR non-LTR retrotransposon (in red) and a putative non-transcribed spacer (NTS) (rest of the sequence). In the short fragment (A), the consensus sequence is shown for specimen no. A7548 and only base changes according to this sequence are shown for the specimen no. A7549. Dots indicate the upper consensus sequence. Sequence of the long fragment (B) was assembled only from specimen no. A7548. (PDF 111 kb) Additional file 7: Figure S3. Karyotypes arranged from Giemsa-stained chromosomes and dual-colour FISH showing 5S and 45S rDNA sites. Giemsa-stained karyotypes (left column) and dual-colour FISH (right column) with 45S rDNA (red, arrows) and 5S rDNA (green, arrowheads) probes on (A,B) *L. costata*, (C,D) *M. guentheri*, (E,F) *N. binotatus*, (G,H) *P. pictilis*, (I,J) *P. brevis*, (K.L) *P. lucidorsum*, (M,N) *S. bolavenensis*, (O,P) *S. corica*, (Q,R) *S. hypsiura*, (S,T) *S. pridii*, (U,V) *S. lendlii*, (W) *S. savona.* The FISH chromosomes were counterstained with DAPI and the images were converted to grayscale. Inset (D) – chromosome pair 18 from *M. guentheri* female showing absence of heterochromatic p-arm in contrast to a single homologue in the male karyotype. Inset (V) depicts the absence of a 45S rDNA site on one homologue in female (pair 3). In *P. brevis* (J), note the syntenical association of both rDNAs on pair 13. Note also the intense size polymorphism in *S. bolavenensis* (pair 1) (N) and *S. corica* (pair 7) (P). Additional polymorphic rDNA sites from the other specimen are boxed for *S. corica* (pairs 7 and 17) (P) and *S. hypsiura* (pair 18) (R). Bar = 10 μm. (ZIP 2482 kb) Additional file 8: Figure S4. Karyotypes of *S. notostigma* after different cytogenetic protocols. (A-D) karyomorph with 44 chromosomes, (E,F) karyomorph with 48 chromosomes. (A,E) conventional Giemsa staining, (C) C-banding, (B,D,F) dual-colour FISH with 45S rDNA (red, arrows) and 5S rDNA (green, arrowheads) probes. Arrangement of st-a chromosome pairs 18 and 19 (E,F) demonstrates a putative origin (centric fusion) of metacentric chromosome pair 2 (A-D). Note also chromosome pairs heterozygous for presence/absence of 45S rDNA site (pair 12) (D) or 5S rDNA site (pair 22) (F). Finally, notice conspicuous regions of constitutive heterochromatin located in centromeres of metacentric pairs 1 and 2 and those located intercalarly on q-arms of sm chromosome pairs 11, 13, 14, 15, 17. Bar = 10 μm. (TIF 540 kb) Additional file 9: Table S4. Distribution of AT- and GC-rich sites and its relation to constitutive heterochromatin and rDNA regions in nemacheilid genomes as inferred from C-banding, DAPI- and CMA3-stainings. Species order reflects their phylogenetic relationships. (XLSX 13 kb) Additional file 10: Figure S5. Mitotic metaphases of selected nemacheilid species after CDD banding. (A) *L. costata,* (B) *P. zonalternans*, (C) *P.* sp., (D) *P. lucidorsum*, (E) *S. fasciolata*, (F) *S. hypsiura*, (G) *S. notostigma* (karyomorph with 48 chromosomes) (H) *S. pridii*, (I) *S. savona*, (J) *S. lendlii*. Pictures were pseudocoloured in red (for CMA_3_) and green (for DAPI). Bar = 10 μm. (TIF 2549 kb) Additional file 11: Figure S6. Karyotypes of *P. zonalternans* and *P. elongata* after Giemsa staining and dual-colour (5S/45S) rDNA FISH. 45S rDNA (red, arrows) and 5S rDNA (green, arrowheads) probes on (A,B) *P. zonalternans* and (C,D) *P. elongata*. Insets – chromosomes showing co-localization of 45S and 5S rDNA – *P. zonalternans*, pair 12 (B); *P. elongata* pairs 4, 12 (D). For clarity, chromosomes are arranged as separated images for each rDNA probe. Note also the heterozygosity for inverted 45S rDNA locus – *P. zonalternans*, pair 10 (B); *P. elongata,* pair 5 (D). The asterisk denotes a missing chromatid in one homologue of chromosome pair 25 in *P. elongata* (D). Bar = 10 μm. (TIF 522 kb) Additional file 12: Figure S7. Mitotic metaphases of selected nemacheilid species after TSA FISH with telomeric (TTAGGG)_n_ probe. (A) *L. costata*, (B) *P. brevis*, (C) *P. lucidorsum,* (D) *S. notostigma* (karyomorph with 48 chromosomes). Chromosomes with the telomeric repeat probe (green) are counterstained with DAPI, pseudocoloured in red for better contrast. Bar = 10 μm. (TIF 785 kb)

## Abbreviations

AT: Adenine-thymine
CMA_3_: Chromomycin A_3_
BSA: Bovine serum albumin
*cyt b*: Cytochrome b
DAPI: 4′, 6-diamidino-2-phenylindole
DAPI/CMA_3_^+/−^: DAPI/CMA_3_ positive/negative signals
FISH: Fluorescence in situ hybridization
FITC: Fluorescein isothiocyanate
GC: Guanine-cytosine
GIRI: Genetic information research institute
het-ITSs: Heterochromatic interstitial telomeric sequences
IAPG: Institute of Animal Physiology and Genetics
IGS: Intergenic spacer
*IRBP*: Interphotoreceptor retinoid-binding protein
ITSs: Interstitial telomeric sequences
LTR: long terminal repeat
NF: Nombre fundamental
NOR: Nucleolar organizer region
NTS: Non-transcribed spacer
PCR: Polymerase chain reaction
*RAG1*: Recombination-activating gene 1
rDNA: Ribosomal DNA
RTEs: Retrotransposable elements
SSC: Standard saline buffer
TEs: Transposable elements
TSA: Tyramid signal amplification
2n: Diploid chromosome number
