# Supplementary material for: Karyotype differentiation in 19 species of river loach fishes (Nemacheilidae, Teleostei): extensive variability associated with rDNA and heterochromatin distribution and its phylogenetic and ecological interpretation
Source: BMC Evol Biol. 2015 Nov 14;15:251. doi: 10.1186/s12862-015-0532-9 (PMC4647339; doi:10.1186/s12862-015-0532-9)
Supplement: Additional file 1: — Supplementary Methods 1. Taxonomic identification of nemacheilids. Supplementary Methods 2. Preparation of chromosomes from regenerating fin tissue. Supplementary Methods 3. PCR conditions of 5S and 45S rDNA amplification. Supplementary Methods 4. PCR conditions of RAG1, IRBP and cyt b amplification. Supplementary Discussion. Possible functional consequences of excessive 5S rDNA copies. (PDF 181 kb) [file 12862_2015_532_MOESM1_ESM.pdf]

Additional file 1

Supplementary Methods

1) Taxonomic identification of nemacheilids

Specimens were identified following:

[1] Bănărescu P, Nalbant TT. A generical classification of Nemacheilinae with description of two new genera (Teleostei: Cypriniformes: Cobitidae). Travaux du Museum d'Histoire Naturelle "Grigore Antipa". 1995;35:429–96.

[2] Bohlen J, Šlechtová V: A new genus and two new species of loaches (Teleostei: Nemacheilidae) from Myanmar. Ichthyol Explor Freshw. 2011;22:1–10.

[3] Bohlen J, Šlechtová, V, Udomritthiruj K. *Schistura hypsiura*, a new species of loach (Cobitoidea: Nemacheilidae) from South-West Myanmar. Raffles Bull Zool. 2014;62:21–7.

[4] Kottelat M. Indochinese Nemacheilines, a revision of nemacheiline loaches (Pisces: Cypriniformes) of Thailand, Burma, Laos, Cambodia and southern Viet Nam. Pfeil, München. 1990;1–262.

[5] Kottelat M. Fishes of Laos. Wildlife Heritage Trust: Colombo; 2001

[6] Menon AGK. The fauna of India and adjacent countries. Pisces. Vol. IV. Teleostei – Cobitoidea. Part 1. Homalopteridae. Zoological Survey of India, Calcutta. 1987;1–260.

[7] Singer RA, Page LM. Revision of the zipper loaches, *Acanthocobitis* and *Paracanthocobitis* (Teleostei: Nemacheilidae), with descriptions of five new species. Copeia. 2015;103:378–401.

[8] Vidthayanon C: *Schistura pridii*, a new nemacheiline loach (Teleostei: Balitoridae) from Upper Chao Phraya drainage, northern Thailand. Ichthyol Explor Freshw. 2003;14:307–10.

2) Preparation of chromosomes from regenerating fin tissue

Briefly, the posterior margin of the caudal fin was cut off and the tissue was used for DNA extraction. Three weeks later, the regenerated tissue of the fin was collected again and subsequently incubated in Ringer solution [9] with combined mitostatic (0.025% colchicine) and hypotonic effect (2h, RT). Next, three rounds of cold fixation with methanol:acetic acid 3:1 (v/v) were performed (25 min at 4°C each). Occasionally, the last fixation step was prolonged overnight. The fixed fin tissue was minced in 50% acetic acid. The resulting suspension was dropped onto pre-heated slides (50°C) and excess removed after 20 sec. This non-invasive technique developed by [10] enabled us to examine very rare and/or very small-sized species included in the study without sacrificing the indviduals.

[9] Ogawa M, Yagasaki M, Yamazaki F: The effect of prolactin on water influx in isolated gills of the goldfish, *Carassius auratus* L. Comp Biochem Physiol. 1973;44A:1177–83.

[10] Völker M, Sonnenberg R, Ráb P, Kullmann H. Karyotype differentiation in *Chromaphyosemion* killifishes (Cyprinodontiformes, Nothobranchiidae). II: cytogenetic and mitochondrial DNA analyses demonstrate karyotype differentiation and its evolutionary direction in *C. riggenbachi*. Cytogenet Genome Res. 2006;115:70–83.

3) PCR conditions of 5S and 45S rDNA amplification

Thermal profiles were based on [11] (for 5S rDNA) and [12] (for 45S rDNA). A 50 µl PCR reaction mix was prepared with 10 µM of each set of primers, 1x Taq buffer with $MgCl_2$, 100 ng of template DNA, 2.5U Taq polymerase Unis (Top Bio, Prague, Czech Republic) and 10 mM of each dNTP (Top Bio). Desired fragments were obtained after 35 cycles of amplification with annealing at 55°C (for 5S rDNA) or 34 cycles with annealing at 53°C (for 45S).

[11] Alves-Costa FA, Martins C, de Matos FDC, Foresti F, Oliveira C, Wasko AP. 5S rDNA characterization in twelve Sciaenidae fish species (Teleostei, Perciformes): Depicting gene diversity and molecular markers. Genet Mol Biol. 2008;31:303–7.

[12] Zhang Q, Cooper RK, Tiersch TR. Chromosomal location of the 28S ribosomal RNA gene of channel catfish by *in situ* polymerase chain reaction. J Fish Biol. 2000;56:388–97.

4) PCR conditions of *RAG1*, *IRBP* and *cyt b* amplification

PCR amplifications of *cyt b*, *IRBP* and *RAG1* were performed in 25 µl reaction volumes of 10 mM Tris-HCl, 50 mM $(NH_4)_2SO_4$, 0.1% of Triton X-100, 1.5 mM $MgCl_2$, 2 mM TMA oxalate (PCR enhancer), containing 5 nmol of each nucleotide, 1.25 U of *Taq* polymerase (all chemicals by Top-Bio) and 12.5 pmol of each primer.

The PCR reaction profile for *cyt b* and *RAG1* (carried out on MJ Research thermocycler) included 5 min of initial denaturation at 95°C, touch-down profile of 1 min at 94°C, 1 min 30 s at 60-55°C (1°C/cycle) and 2 min at 72°C, followed by 30 cycles with annealing temperature held at 54°C. The reaction was completed by final extension at 72°C for 7 min. The amplification of *IRBP* consisted of 2 min of initial denaturation at 95°C followed by 35 cycles each including denaturation step at 94°C for 30s, a primer annealing step at 59°C and 54°C for primer combinations 101F+1162R and 109F and 1001R,

respectively for 30s, and elongation at 72°C for 45s. The PCR was completed by a final elongation step of 5 min at 72°C.

Supplementary Discussion

Possible functional consequences of excessive 5S rDNA copies

If so many 5S rDNA copies are present: how is expression regulated in an unbalanced ratio with 45S rDNA clusters? Although retrotransposed 5S rDNA was proven to stay potentially functionable [13], there is a high probability that FISH could also label some of the pseudogenic variants, commonly occuring among different fish species [14]. Alternatively, centromeric satellite sequences derived from 5S rDNA have been reported in some fishes [15].

[13] Drouin G: Expressed retrotransposed 5S rRNA genes in the mouse and rat genomes. Genome. 2000;43:213-5.

[14] Martins C, Wasko AP: Organization and evolution of 5S ribosomal DNA in the fish genome. In: Williams CR, editor. Focus on genome research. Hauppauge: Nova Science Publishers; 2004. p.335–63.

[15] Martins C, Ferreira IA, Oliveira C, Foresti F, Galetti PM Jr. A tandemly repetitive centromeric DNA sequence of the fish *Hoplias malabaricus* (Characiformes: Erythrinidae) is derived from 5S rDNA. Genetica. 2006;127:133-41.
