# Supplementary figures and images for: Karyotype differentiation in 19 species of river loach fishes (Nemacheilidae, Teleostei): extensive variability associated with rDNA and heterochromatin distribution and its phylogenetic and ecological interpretation
Source: BMC Evol Biol. 2015 Nov 14;15:251. doi: 10.1186/s12862-015-0532-9 (PMC4647339; doi:10.1186/s12862-015-0532-9)

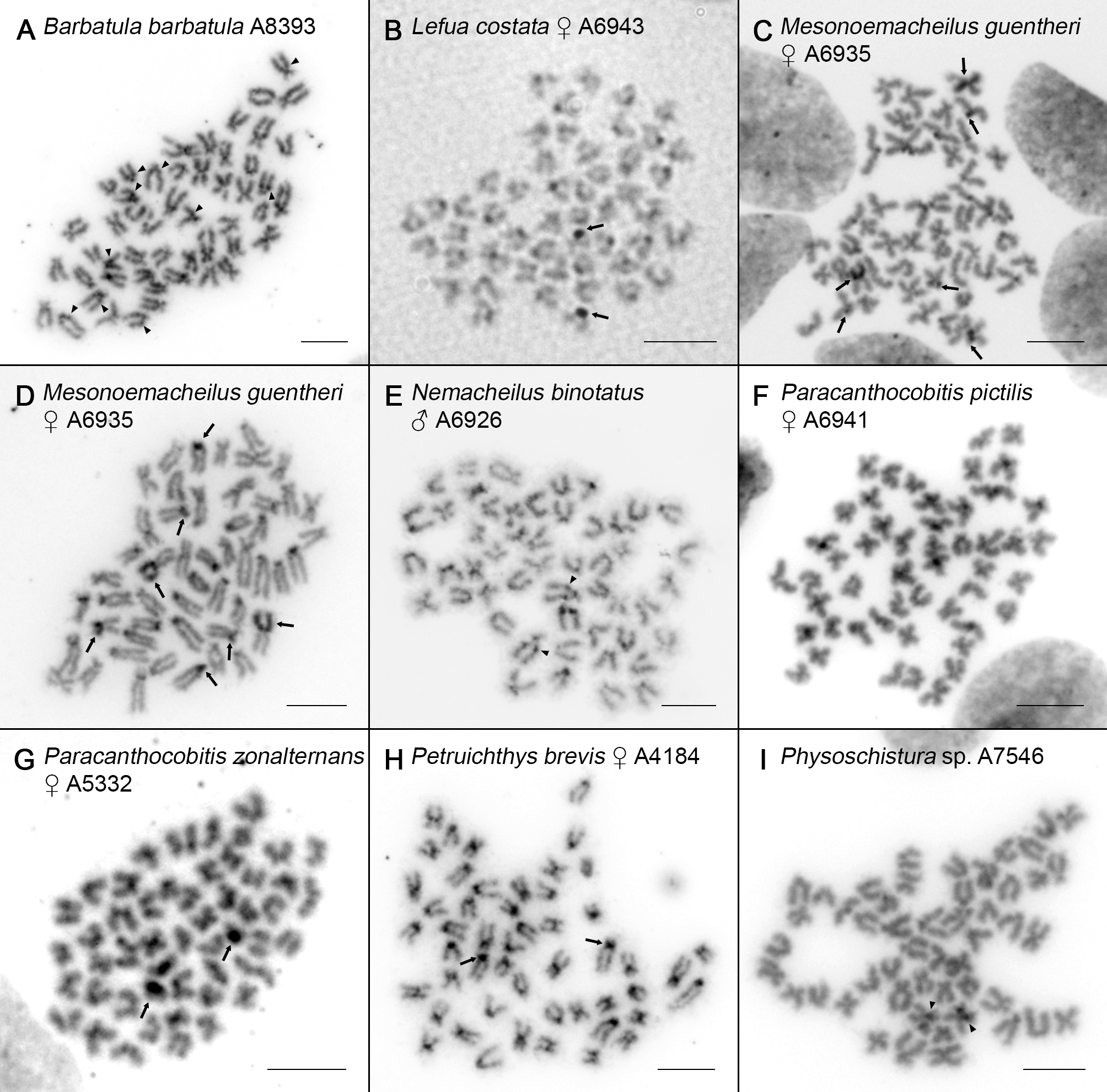

Supplement: Additional file 2: Figure S1. — Mitotic metaphases of selected nemacheilid species after C-banding or DAPI-staining. (A,D,C,F.G,H,I,J,M,O,P,Q) DAPI staining; (D,E,K,L,N) C-banding improved with DAPI counterstaining. Metaphases from both methods are converted to inverted pictures. (A) B. barbatula, (B) L. costata, (C,D) M. guentheri (E) N. binotatus, (F) P. pictilis, (G) P. zonalternans, (H) P. brevis, (I) P. sp., (J) P. elongata, (K) P. lucidorsum, (L) S. bolavenensis, (M) S. corica, (N) S. hypsiura, (O) S. pridii, (P) S. savona, (Q) S. lendlii. Arrows depicts whole-armed heterochromatin, arrowheads denote interstitial heterochromatin, the asterisk indicates C-positive NORs (as rare feature among species under study). For comparison of banding patterns between both methods, see pics. C and D. Note that several species share marked interstitial heterochromatic sites indicating the remnants of putative chromosomal rearrangements (e.g., pericentric inversion) (A,E,L,M,Q). Of particular interest are the completely heterochromatic arms in m-sm chromosomes occuring in a subset of species (C,D,L,M,P,Q). Note also heterochromaic p-arms in some st-a chromosomes (C,D,E,O,P). Bar = 10 μm. (ZIP 4276 kb) [file 12862_2015_532_MOESM2_ESM.zip › 12862_2015_532_add2/Additional_file_2_Fig_S1_C_banding_part_1.tif]

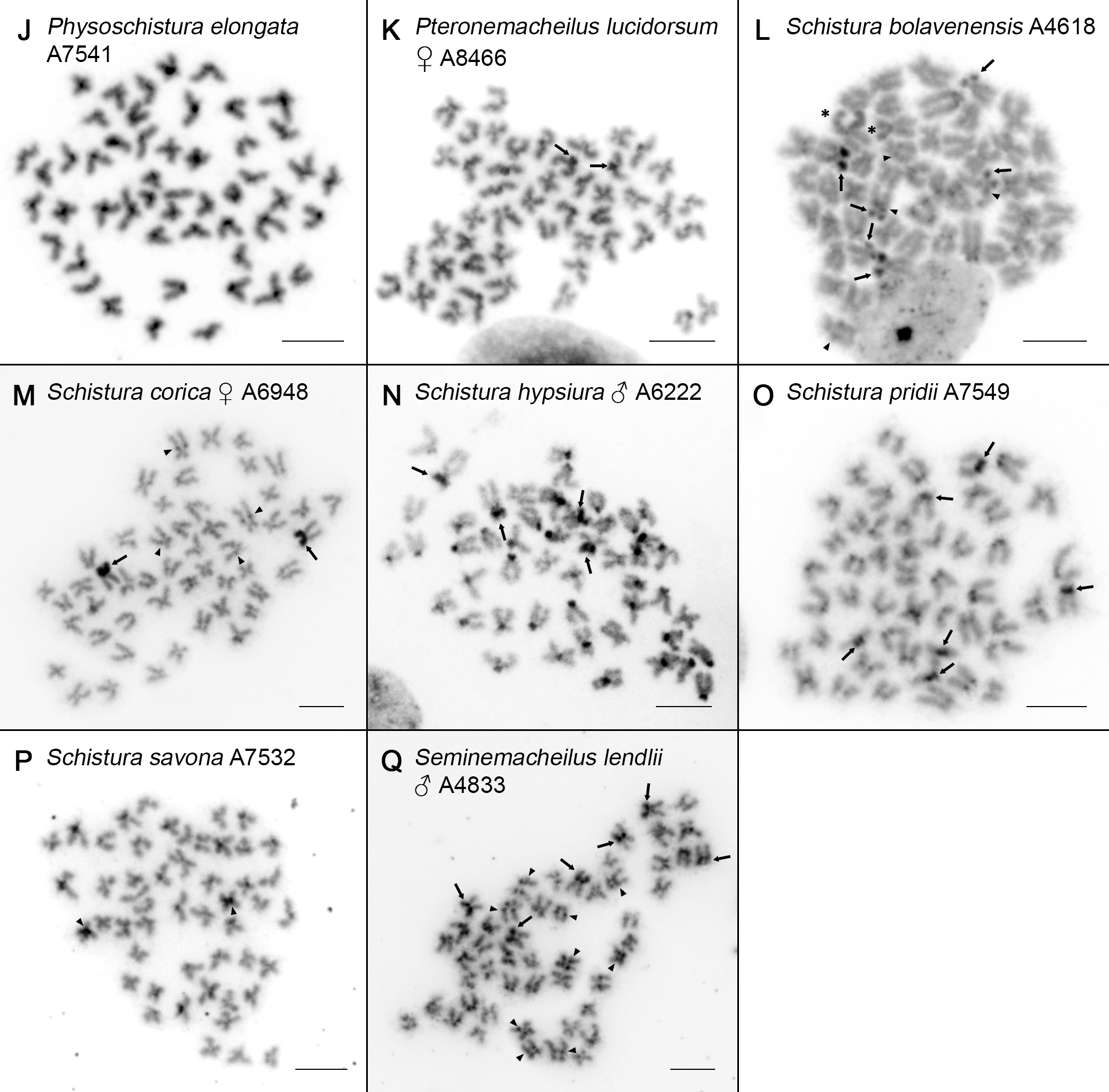

Supplement: Additional file 2: Figure S1. — Mitotic metaphases of selected nemacheilid species after C-banding or DAPI-staining. (A,D,C,F.G,H,I,J,M,O,P,Q) DAPI staining; (D,E,K,L,N) C-banding improved with DAPI counterstaining. Metaphases from both methods are converted to inverted pictures. (A) B. barbatula, (B) L. costata, (C,D) M. guentheri (E) N. binotatus, (F) P. pictilis, (G) P. zonalternans, (H) P. brevis, (I) P. sp., (J) P. elongata, (K) P. lucidorsum, (L) S. bolavenensis, (M) S. corica, (N) S. hypsiura, (O) S. pridii, (P) S. savona, (Q) S. lendlii. Arrows depicts whole-armed heterochromatin, arrowheads denote interstitial heterochromatin, the asterisk indicates C-positive NORs (as rare feature among species under study). For comparison of banding patterns between both methods, see pics. C and D. Note that several species share marked interstitial heterochromatic sites indicating the remnants of putative chromosomal rearrangements (e.g., pericentric inversion) (A,E,L,M,Q). Of particular interest are the completely heterochromatic arms in m-sm chromosomes occuring in a subset of species (C,D,L,M,P,Q). Note also heterochromaic p-arms in some st-a chromosomes (C,D,E,O,P). Bar = 10 μm. (ZIP 4276 kb) [file 12862_2015_532_MOESM2_ESM.zip › 12862_2015_532_add2/Additional_file_2_Fig_S1_C_banding_part_2.tif]

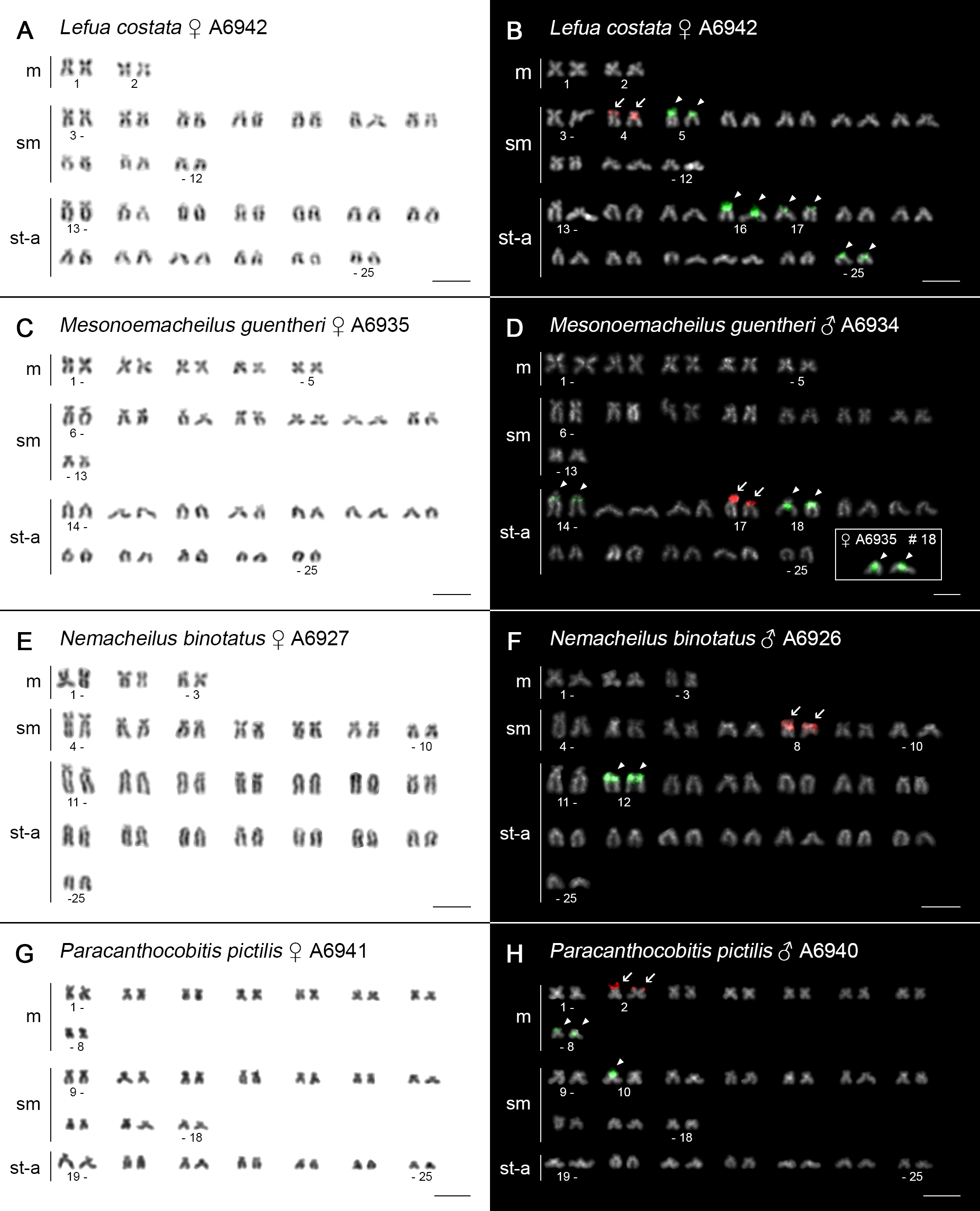

Supplement: Additional file 7: Figure S3. — Karyotypes arranged from Giemsa-stained chromosomes and dual-colour FISH showing 5S and 45S rDNA sites. Giemsa-stained karyotypes (left column) and dual-colour FISH (right column) with 45S rDNA (red, arrows) and 5S rDNA (green, arrowheads) probes on (A,B) L. costata, (C,D) M. guentheri, (E,F) N. binotatus, (G,H) P. pictilis, (I,J) P. brevis, (K.L) P. lucidorsum, (M,N) S. bolavenensis, (O,P) S. corica, (Q,R) S. hypsiura, (S,T) S. pridii, (U,V) S. lendlii, (W) S. savona. The FISH chromosomes were counterstained with DAPI and the images were converted to grayscale. Inset (D) – chromosome pair 18 from M. guentheri female showing absence of heterochromatic p-arm in contrast to a single homologue in the male karyotype. Inset (V) depicts the absence of a 45S rDNA site on one homologue in female (pair 3). In P. brevis (J), note the syntenical association of both rDNAs on pair 13. Note also the intense size polymorphism in S. bolavenensis (pair 1) (N) and S. corica (pair 7) (P). Additional polymorphic rDNA sites from the other specimen are boxed for S. corica (pairs 7 and 17) (P) and S. hypsiura (pair 18) (R). Bar = 10 μm. (ZIP 2482 kb) [file 12862_2015_532_MOESM7_ESM.zip › 12862_2015_532_add7/Additional_file_7_Fig_S3_Karyotypes_part_1.tif]

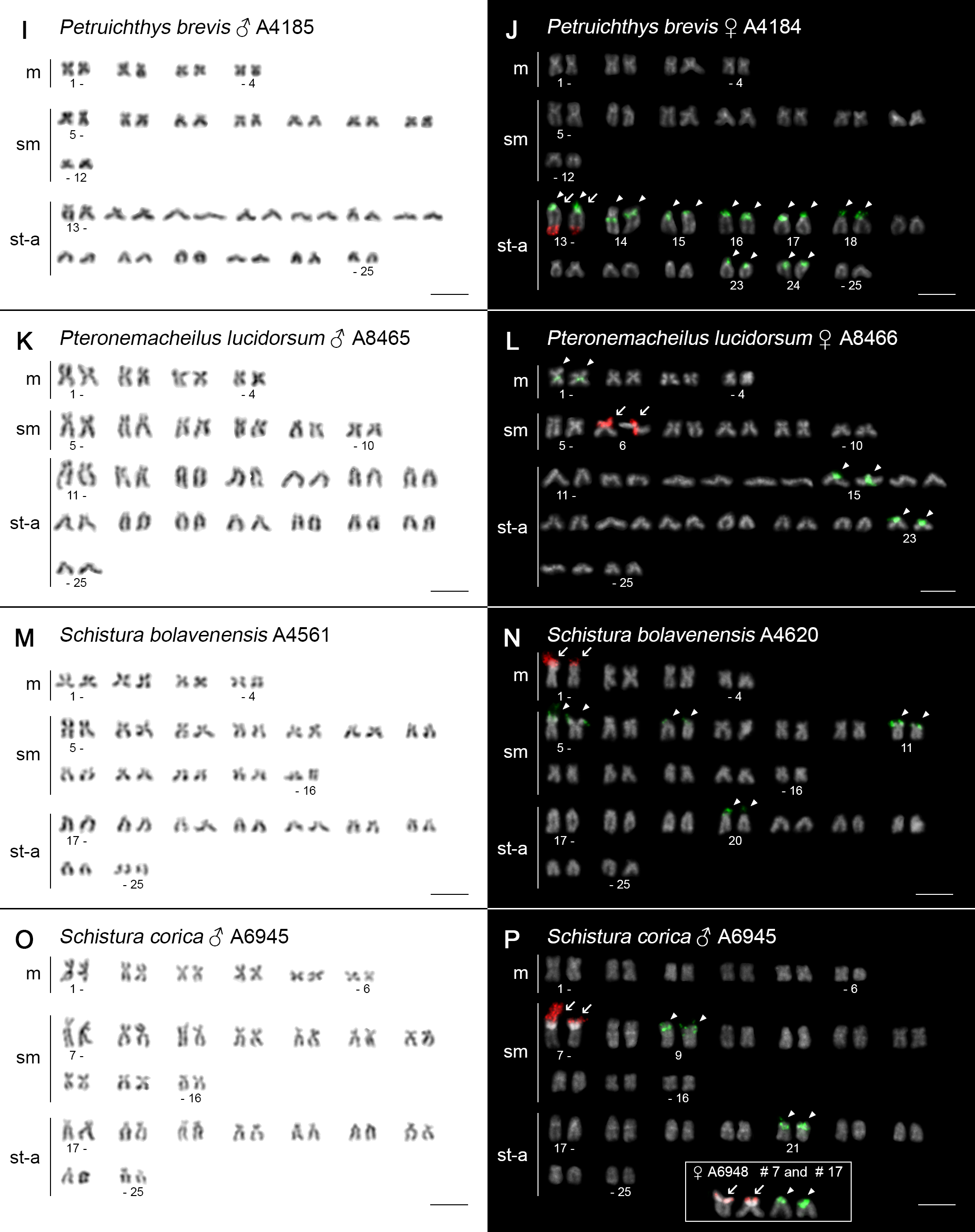

Supplement: Additional file 7: Figure S3. — Karyotypes arranged from Giemsa-stained chromosomes and dual-colour FISH showing 5S and 45S rDNA sites. Giemsa-stained karyotypes (left column) and dual-colour FISH (right column) with 45S rDNA (red, arrows) and 5S rDNA (green, arrowheads) probes on (A,B) L. costata, (C,D) M. guentheri, (E,F) N. binotatus, (G,H) P. pictilis, (I,J) P. brevis, (K.L) P. lucidorsum, (M,N) S. bolavenensis, (O,P) S. corica, (Q,R) S. hypsiura, (S,T) S. pridii, (U,V) S. lendlii, (W) S. savona. The FISH chromosomes were counterstained with DAPI and the images were converted to grayscale. Inset (D) – chromosome pair 18 from M. guentheri female showing absence of heterochromatic p-arm in contrast to a single homologue in the male karyotype. Inset (V) depicts the absence of a 45S rDNA site on one homologue in female (pair 3). In P. brevis (J), note the syntenical association of both rDNAs on pair 13. Note also the intense size polymorphism in S. bolavenensis (pair 1) (N) and S. corica (pair 7) (P). Additional polymorphic rDNA sites from the other specimen are boxed for S. corica (pairs 7 and 17) (P) and S. hypsiura (pair 18) (R). Bar = 10 μm. (ZIP 2482 kb) [file 12862_2015_532_MOESM7_ESM.zip › 12862_2015_532_add7/Additional_file_7_Fig_S3_Karyotypes_part_2.tif]

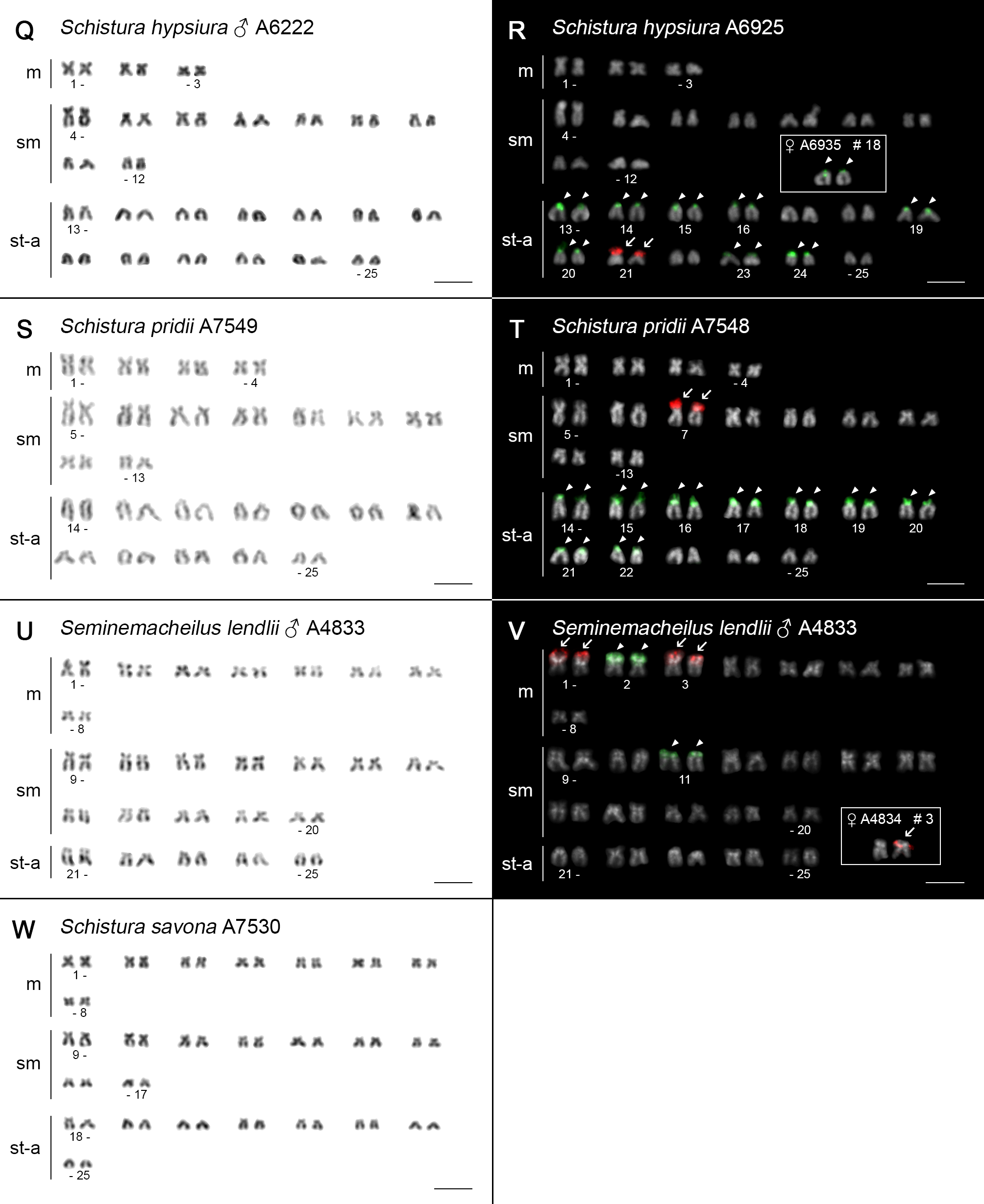

Supplement: Additional file 7: Figure S3. — Karyotypes arranged from Giemsa-stained chromosomes and dual-colour FISH showing 5S and 45S rDNA sites. Giemsa-stained karyotypes (left column) and dual-colour FISH (right column) with 45S rDNA (red, arrows) and 5S rDNA (green, arrowheads) probes on (A,B) L. costata, (C,D) M. guentheri, (E,F) N. binotatus, (G,H) P. pictilis, (I,J) P. brevis, (K.L) P. lucidorsum, (M,N) S. bolavenensis, (O,P) S. corica, (Q,R) S. hypsiura, (S,T) S. pridii, (U,V) S. lendlii, (W) S. savona. The FISH chromosomes were counterstained with DAPI and the images were converted to grayscale. Inset (D) – chromosome pair 18 from M. guentheri female showing absence of heterochromatic p-arm in contrast to a single homologue in the male karyotype. Inset (V) depicts the absence of a 45S rDNA site on one homologue in female (pair 3). In P. brevis (J), note the syntenical association of both rDNAs on pair 13. Note also the intense size polymorphism in S. bolavenensis (pair 1) (N) and S. corica (pair 7) (P). Additional polymorphic rDNA sites from the other specimen are boxed for S. corica (pairs 7 and 17) (P) and S. hypsiura (pair 18) (R). Bar = 10 μm. (ZIP 2482 kb) [file 12862_2015_532_MOESM7_ESM.zip › 12862_2015_532_add7/Additional_file_7_Fig_S3_Karyotypes_part_3.tif]

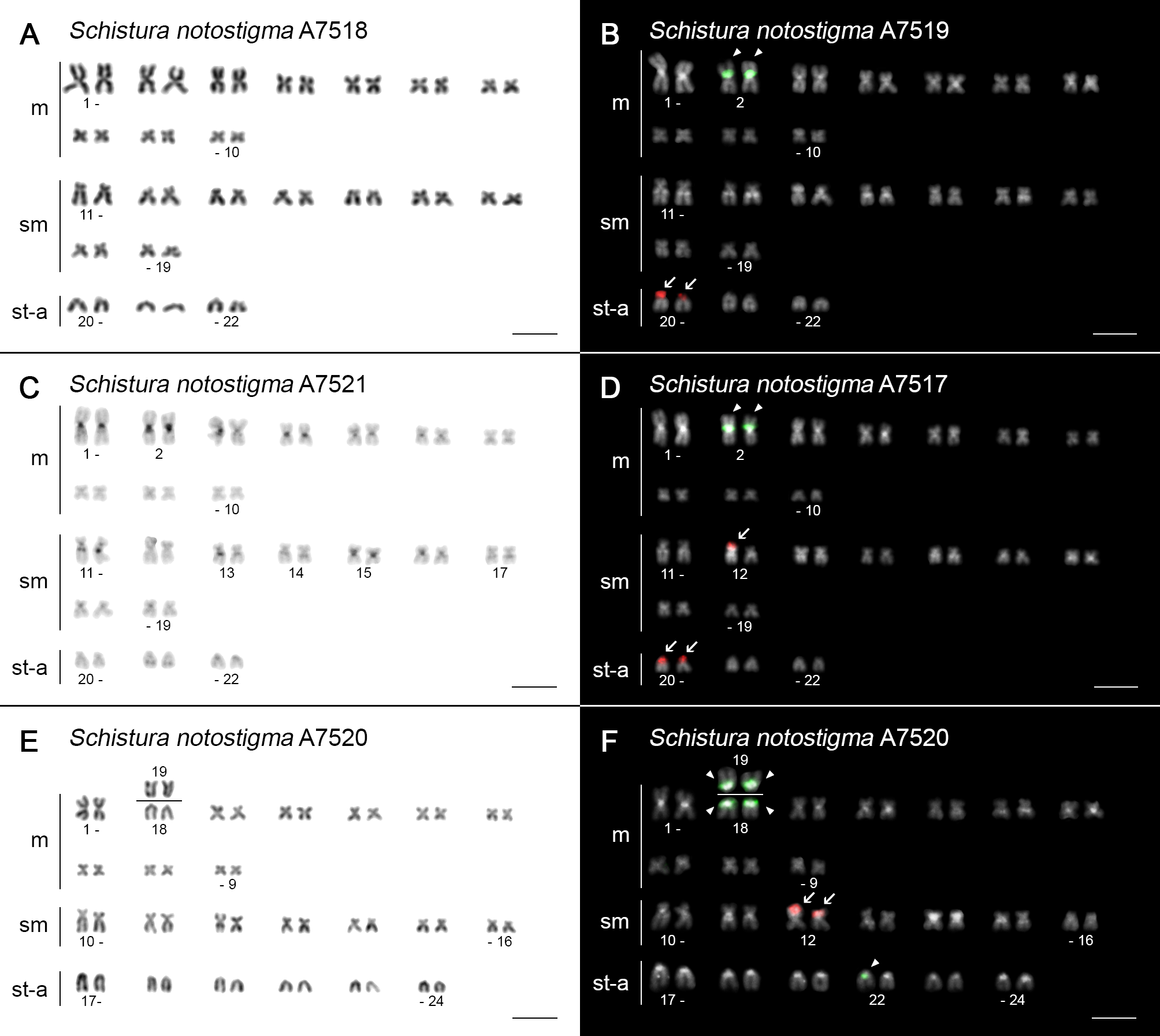

Supplement: Additional file 8: Figure S4. — Karyotypes of S. notostigma after different cytogenetic protocols. (A-D) karyomorph with 44 chromosomes, (E,F) karyomorph with 48 chromosomes. (A,E) conventional Giemsa staining, (C) C-banding, (B,D,F) dual-colour FISH with 45S rDNA (red, arrows) and 5S rDNA (green, arrowheads) probes. Arrangement of st-a chromosome pairs 18 and 19 (E,F) demonstrates a putative origin (centric fusion) of metacentric chromosome pair 2 (A-D). Note also chromosome pairs heterozygous for presence/absence of 45S rDNA site (pair 12) (D) or 5S rDNA site (pair 22) (F). Finally, notice conspicuous regions of constitutive heterochromatin located in centromeres of metacentric pairs 1 and 2 and those located intercalarly on q-arms of sm chromosome pairs 11, 13, 14, 15, 17. Bar = 10 μm. (TIF 540 kb) [file 12862_2015_532_MOESM8_ESM.tif]

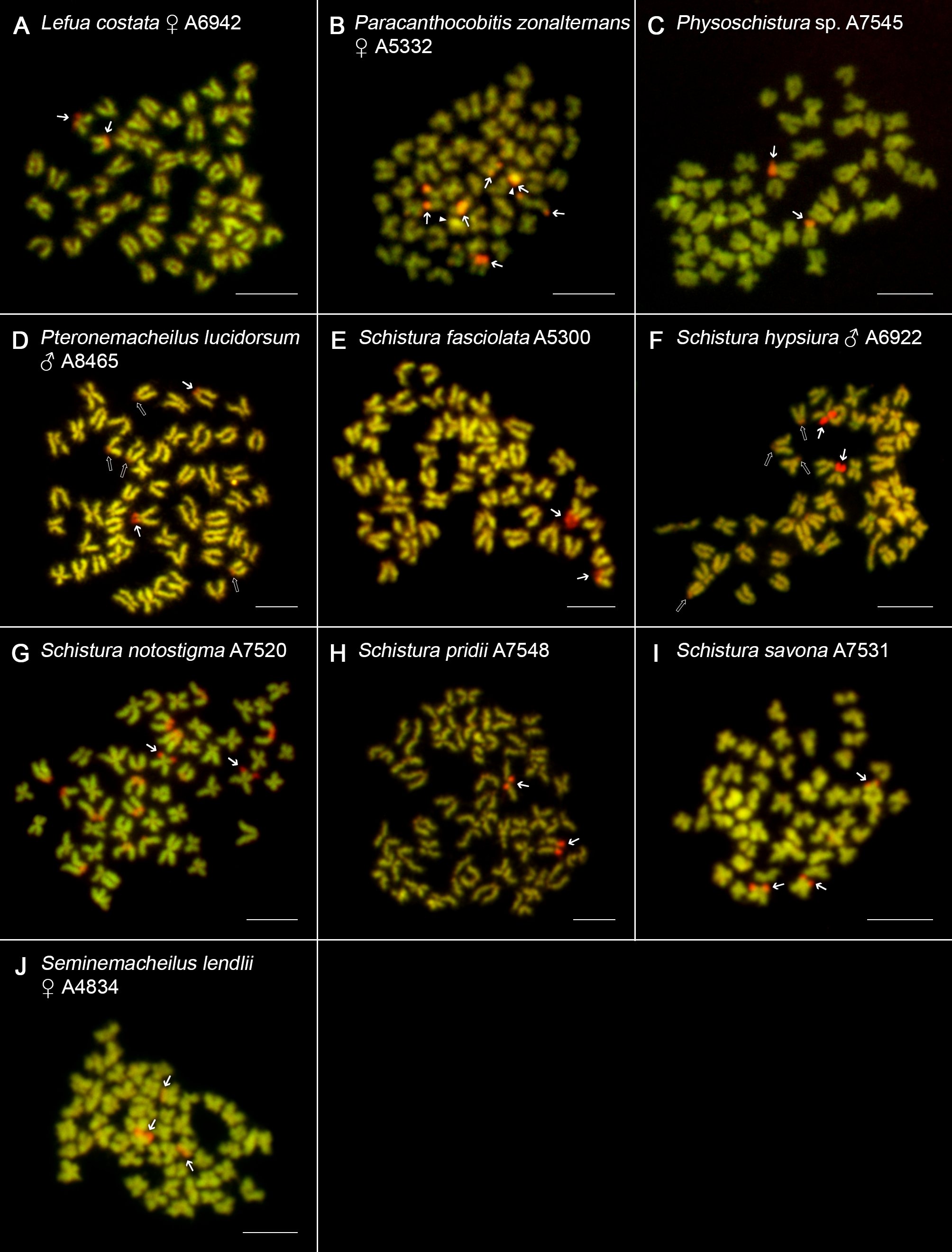

Supplement: Additional file 10: Figure S5. — Mitotic metaphases of selected nemacheilid species after CDD banding. (A) L. costata, (B) P. zonalternans, (C) P. sp., (D) P. lucidorsum, (E) S. fasciolata, (F) S. hypsiura, (G) S. notostigma (karyomorph with 48 chromosomes) (H) S. pridii, (I) S. savona, (J) S. lendlii. Pictures were pseudocoloured in red (for CMA3) and green (for DAPI). Bar = 10 μm. (TIF 2549 kb) [file 12862_2015_532_MOESM10_ESM.tif]

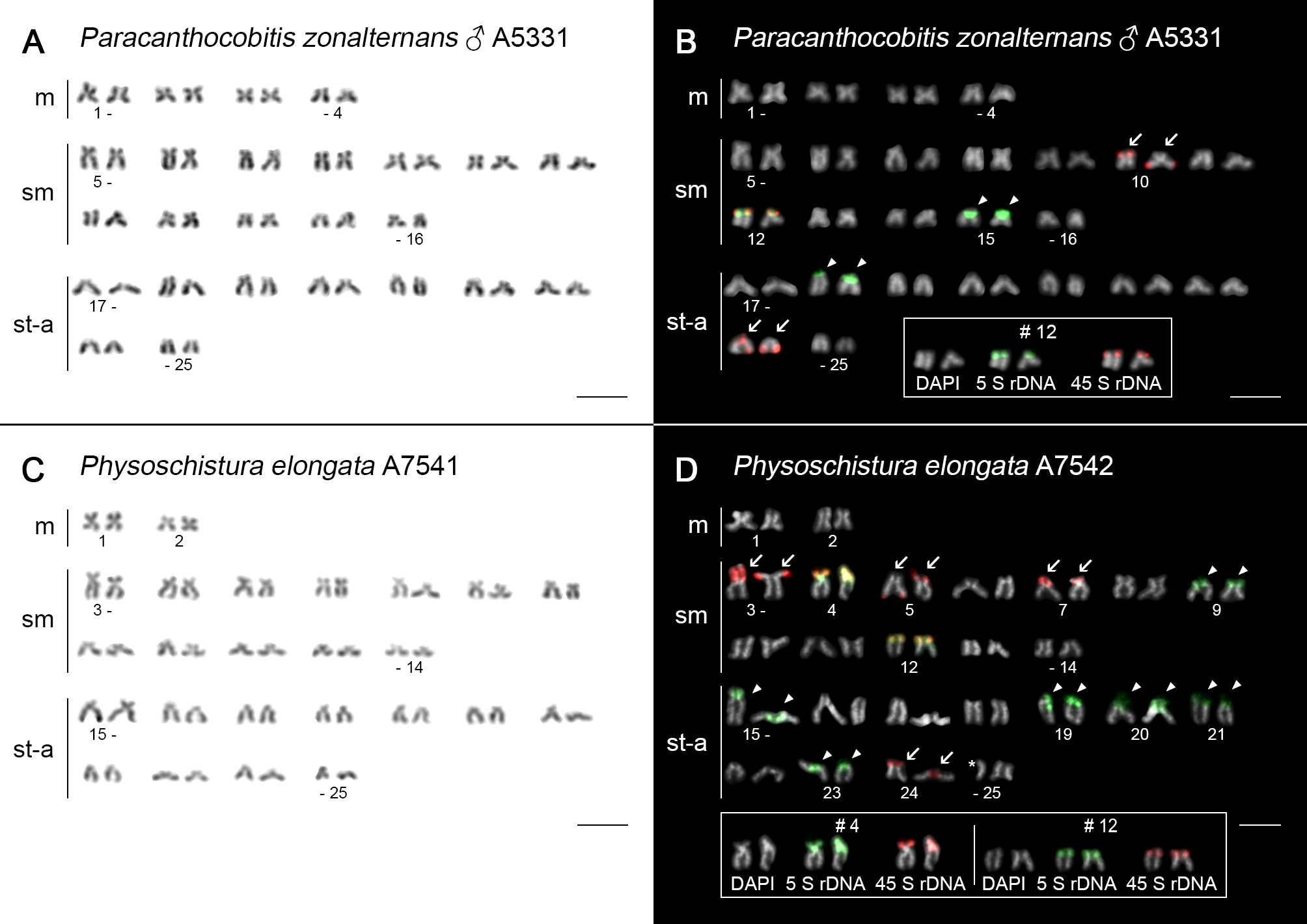

Supplement: Additional file 11: Figure S6. — Karyotypes of P. zonalternans and P. elongata after Giemsa staining and dual-colour (5S/45S) rDNA FISH. 45S rDNA (red, arrows) and 5S rDNA (green, arrowheads) probes on (A,B) P. zonalternans and (C,D) P. elongata. Insets – chromosomes showing co-localization of 45S and 5S rDNA – P. zonalternans, pair 12 (B); P. elongata pairs 4, 12 (D). For clarity, chromosomes are arranged as separated images for each rDNA probe. Note also the heterozygosity for inverted 45S rDNA locus – P. zonalternans, pair 10 (B); P. elongata, pair 5 (D). The asterisk denotes a missing chromatid in one homologue of chromosome pair 25 in P. elongata (D). Bar = 10 μm. (TIF 522 kb) [file 12862_2015_532_MOESM11_ESM.tif]

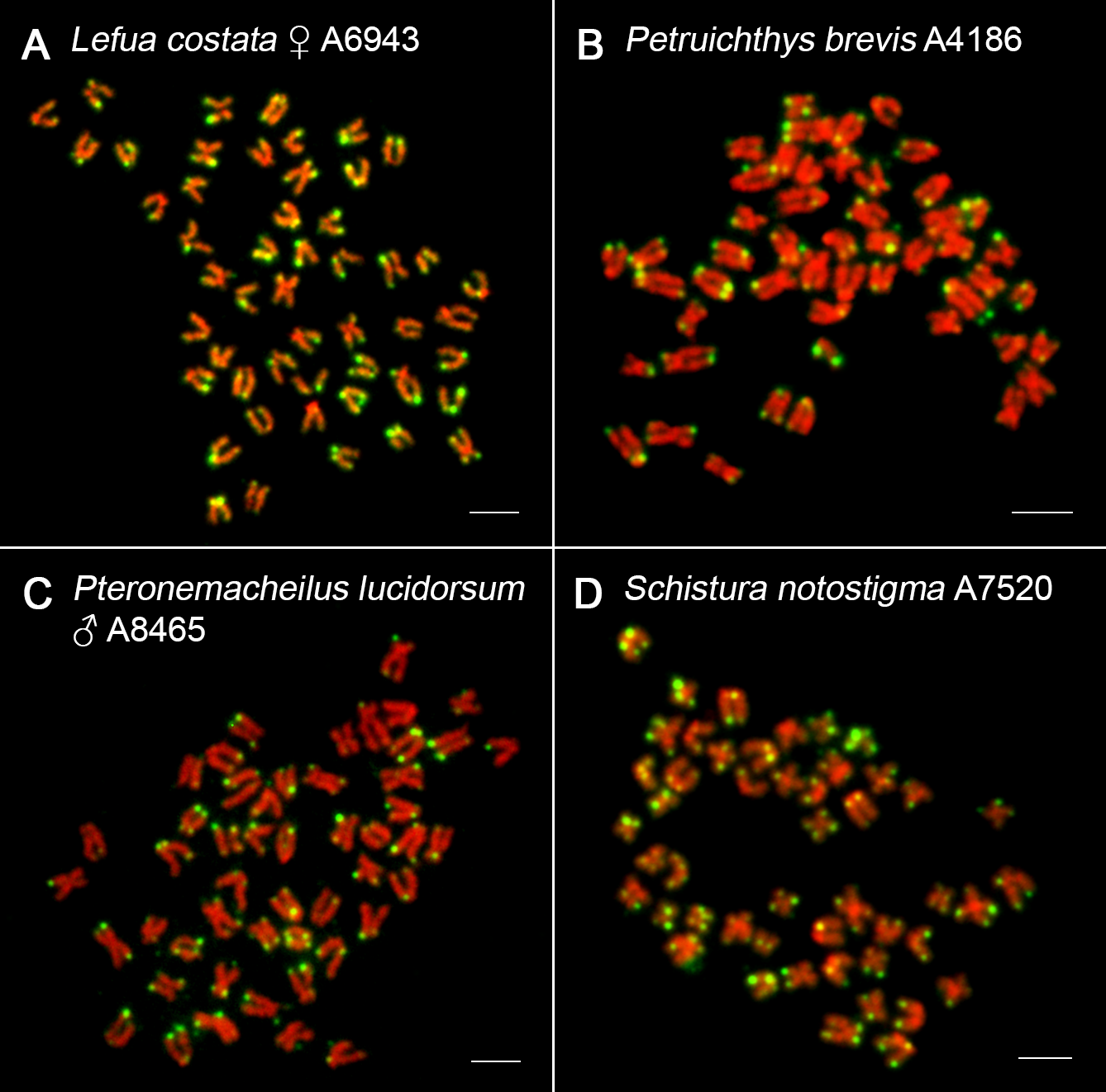

Supplement: Additional file 12: Figure S7. — Mitotic metaphases of selected nemacheilid species after TSA FISH with telomeric (TTAGGG)n probe. (A) L. costata, (B) P. brevis, (C) P. lucidorsum, (D) S. notostigma (karyomorph with 48 chromosomes). Chromosomes with the telomeric repeat probe (green) are counterstained with DAPI, pseudocoloured in red for better contrast. Bar = 10 μm. (TIF 785 kb) [file 12862_2015_532_MOESM12_ESM.tif]
