# Supplementary material for: Karyotype differentiation in 19 species of river loach fishes (Nemacheilidae, Teleostei): extensive variability associated with rDNA and heterochromatin distribution and its phylogenetic and ecological interpretation
Source: BMC Evol Biol. 2015 Nov 14;15:251. doi: 10.1186/s12862-015-0532-9 (PMC4647339; doi:10.1186/s12862-015-0532-9)
Supplement: Additional file 3: Table S1. — Primer sequences used in this study. (PDF 221 kb) [file 12862_2015_532_MOESM3_ESM.pdf]

***Cytochrome b***

| Primer Name | Sequence 5'-3' | Direction | Reference |
|---|---|---|---|
| Glu L.Ca14337-14359: | GAA GAA CCA CCG TTG TTA TTC AA | F | [16] |
| Thr H.Ca15568-15548: | ACC TCC RAT CTY CGG ATT ACA | R | [16] |

***RAG 1***

| Primer Name | Sequence 5'-3' | Direction | Reference |
|---|---|---|---|
| RAG-1F | AGC TGT AGT CAG TAY CAC AAR ATG | F | [17] |
| RAG-Rv1 | TCC TGR AAG ATY TTG TAG AA | R | [18] |

***IRBP***

| Primer Name | Sequence 5'-3' | Direction | Reference |
|---|---|---|---|
| IRBP 101F | TCM TGG ACA AYT ACT GCT CAC C | F | [19] |
| IRBP 109F | AAC TAC TGC TCR CCA GAA AAR C | F | [19] |
| IRBP 1001R | GGA AAT GCA TAG TTG TCT GCA A | R | [19] |
| IRBP 1162R | TGG TGG WCT TYA GGC ACT TGT | R | [19] |

**5S rRNA**

| Primer Name | Sequence 5'-3' | Direction | Reference |
|---|---|---|---|
| 5SA | TAC GCC CGA TCT CGT CCG ATC | F | [20] |
| 5SB | CAG GCT GGT ATG GCC GTA AGC | R | [20] |

**28S rRNA**

| Primer Name | Sequence 5'-3' | Direction | Reference |
|---|---|---|---|
| 28SA | AAA CTC TGG TGG AGG TCC GT | F | [21] |
| 28SB | CTT ACC AAA AGT GGC CCA CTA | R | [21] |

[16] Šlechtová V, Bohlen J, Freyhof J, Ráb P. Molecular phylogeny of the Southeast Asian freshwater fish family Botiidae (Teleostei: Cobitoidea) and the origin of polyploidy in their evolution. Mol Phylogenet Evol. 2006;39:529-41.

[17] Quenouille B, Bermingham E, Planes S: Molecular systematics of the damselfishes (Teleostei : Pomacentridae): Bayesian phylogenetic analyses of mitochondrial and nuclear DNA sequences. Mol Phylogenet Evol. 2004;31:66-88.

[18] Šlechtová V, Bohlen J, Tan HH: Families of Cobitoidea (Teleostei; Cypriniformes) as revealed from nuclear genetic data and the position of the mysterious genera *Barbucca*, *Psilorhynchus*, *Serpenticobitis* and *Vaillantella*. Mol Phylogenet Evol. 2007;44:1358-65.

[19] Chen, W-J, Miya M, Saitoh K, Mayden RL: Phylogenetic utility of two existing and four novel nuclear gene loci in reconstructing Tree of Life of ray-finned fishes: The order Cypriniformes (Ostariophysi) as a case study. Gene. 2008;423:125-34.

[20] Martins C, Galetti PM: Chromosomal localization of 5S rDNA genes in *Leporinus* fish (Anostomidae, Characiformes). Chromosome Res. 1999;7:363-7.

[21] Naito E, Dewa K, Yamanouchi H, Kominami R: Ribosomal ribonucleic acid (rRNA) gene typing for species identification. Journal of Forensic Science. 1992;37:396–403.
