# Supplementary material for: Karyotype differentiation in 19 species of river loach fishes (Nemacheilidae, Teleostei): extensive variability associated with rDNA and heterochromatin distribution and its phylogenetic and ecological interpretation
Source: BMC Evol Biol. 2015 Nov 14;15:251. doi: 10.1186/s12862-015-0532-9 (PMC4647339; doi:10.1186/s12862-015-0532-9)
Supplement: Additional file 6: Figure S2. — Sequence alignment of cloned 5S rDNA fragments from S. pridii. Nucleotide sequences (5′-3′) obtained from both specimens (A7548, A7549) corresponding to the short (A) and long (B) variant of 5S rDNA, containing partial 5S rDNA coding sequence (green), partial sequence of L1-2_DR non-LTR retrotransposon (in red) and a putative non-transcribed spacer (NTS) (rest of the sequence). In the short fragment (A), the consensus sequence is shown for specimen no. A7548 and only base changes according to this sequence are shown for the specimen no. A7549. Dots indicate the upper consensus sequence. Sequence of the long fragment (B) was assembled only from specimen no. A7548. (PDF 111 kb) [file 12862_2015_532_MOESM6_ESM.pdf]

A

```
Schistura pridii A7548 short  GTTCGGGCTG GTTAGTACTT GGATGGGAGA CCGCCTGGGA ATACCAGGTG CTGTAAGCCT TTATCATTTT
Schistura pridii A7549        .......... .......... .......... .....Y.... ..R....... .......... ..........

Schistura pridii A7548 short  TGGCTTTCCT TGATTTCGTT TTCAACTGAA TTTTTTAAAG AGCCTGCAGG TCACCGCCTC TTTCGCTTAC
Schistura pridii A7549        .......... .......... .......... .......... .......... .......... ..........

Schistura pridii A7548 short  GGCCATACCA GCCTGA
Schistura pridii A7549        .......... ......
```

B

```
Schistura pridii A7548  GYTCGGGCTG GTYAGTRCTT GGATGGGAGA CCRCCTGGGA ATACYRGGTG CTGTAAGCCT TTATCATTTY

Schistura pridii A7548  TGGCWTTCCT TGATTTCGTT TTCAAYTKAT GCCMTTCGCC TACAGGCTCA GTKTTMATTT WTTCATCTCC

Schistura pridii A7548  ATGCCTAGAC AGCCCAGTGA CTGTTGCTGA RCGAWGTGAG GCTTTTCAAA AATCAACGAA ATSAAAGGAT

Schistura pridii A7548  GAAAGGCATC ATTCCGACTW WGGAWAAACK ARTSGGATAC GCAAAGAAYR AAAAACATKW AAAATAATCT

Schistura pridii A7548  AAAACKCAGT TRTGCTCGCC GGCGTCATTC TSRASCGGGC AAGTAAACGG GACATCTGAC GARTGAGAAC

Schistura pridii A7548  CMTCAWGGGA GTCCCAGGTR AAAGTAAMAT KTTTCTTCCT AATATTAAGC CTGCTCTTGG GACATTCCAA

Schistura pridii A7548  AMAGAAACCC AAGCAGTGGC WTGAAGGGGA AGAGTCGCRT GGTAGCCGTC CAGCTCTTGG CTAGCTAGGA

Schistura pridii A7548  WTTTTTAAAR AKCCTGCAGG TCACMGCCTC TTTCGCTTAC GGCCATACCA GCCTGA
```
